# Mathematical analysis and optimal control interventions for sex structured syphilis model with three stages of infection and loss of immunity

**DOI:** 10.1186/s13662-021-03432-7

**Published:** 2021-06-11

**Authors:** Abdulfatai Atte Momoh, Yusuf Bala, Dekera Jacob Washachi, Dione Déthié

**Affiliations:** 1 Department of Mathematics, Modibbo Adama University of Technology, Yola, Nigeria; 2 Cheikh Anta Diop University, Dakar, Senegal

**Keywords:** 92D40, 93A30, Stability, Hamiltonian, Transmission, Equilibrium states, Epidemiology, Invariant region

## Abstract

In this study, we develop a nonlinear ordinary differential equation to study the dynamics of syphilis transmission incorporating controls, namely prevention and treatment of the infected males and females. We obtain syphilis-free equilibrium (SFE) and syphilis-present equilibrium (SPE). We obtain the basic reproduction number, which can be used to control the transmission of the disease, and thus establish the conditions for local and global stability of the syphilis-free equilibrium. The stability results show that the model is locally asymptotically stable if the Routh–Hurwitz criteria are satisfied and globally asymptotically stable. The bifurcation analysis result reveals that the model exhibits backward bifurcation. We adopted Pontryagin’s maximum principle to determine the optimality system for the syphilis model, which was solved numerically to show that syphilis transmission can be optimally best control using a combination of condoms usage and treatment in the primary stage of infection in both infected male and female populations.

## Introduction

Syphilis is one of the infectious diseases, most commonly caused by sexual contact. Spirochete Treponema pallidum is a spiral-shaped bacteria that causes syphilis [23]. When a person contracts syphilis, he or she develops sores, blisters, or ulcers on his genitals anus (bottom), or mouth [2]. The disease is largely transmitted from one person to another when a susceptible person has sex without condoms or shares sex toys with an infected person [33]. Even though the disease is spread from sores, most of those sores disappeared without being recognized [33]. There are three different stages in syphilis disease: primary stage, secondary stage, and latent stage. At the primary stage the sign may appear as a solitary, painless chancre at the site of inoculation. However, the primary chancre may disappear without being noticed by infected persons. If the disease is left untreated at the primary stage, then it would advance to the secondary stage. The symptoms at this stage are more visible. It includes mucocutaneous lesions affecting both skins, mucous membranes, and lymph nodes [44]. Mostly, at the secondary stage, there are usually rashes on the palms and sole of an infected person, and it can imitate other infectious and noninfectious conditions. It is important to state that the rashes on the palm and foot of an infected person at secondary syphilis stage may also disappear even without treatment. If the disease is left untreated, then the infected person progresses to the latent stage [44]. The latent syphilis is asymptotic, described by positive syphilis serology without any clinical manifestations [44]. At this stage, syphilis is often categorized in to two parts, early latent syphilis and late latent syphilis. The early latent syphilis is characterized by an infection, which is less than two years, whereas the late latent syphilis, on the other hand, is an infection of the disease for two years or beyond [44]. Transmission of syphilis occurs during primary, secondary, or early latent syphilis; moreover, mother-to-child transmission has been observed after several years of infection with syphilis, especially in an untreated cases [44]. According to World Health Organization (WHO), an estimates of 36.4 million syphilis infection cases were recorded worldwide [43]. About 90% of new syphilis cases are found in resource-limited countries out of an estimated population of over 12 million new syphilis infections recorded every year in the world [13, 14]. WHO reported that there are 3.4 million annual new cases of syphilis in the African region among people aged 15–49 years [43]. The global incidence rate of syphilis was 1.5 cases per 1000 females and 1.5 per 1000 males in 2012. It is estimated that the global prevalence of syphilis is at 0.5% among females and 0.5% among males aged 15–49 years, with the highest prevalence in the WHO African Region [44]. In Nigeria the prevalence of syphilis is estimated to be between 1.3 million and 2.8 million (0.7–1.5%) [17, 39]. There were 6498 deaths as a result of syphilis infection, 4149 males and 2349 females in the United States of America from 1968 to 2015. Adults now rarely die due to syphilis [31]. About $492\mbox{,}000$ infants die each year due to syphilis infection from congenital syphilis in sub-Saharan Africa [30]. In Nigeria, neonatal death rate per 1000 is estimated at 34.1 [21]. Despite the introduction of penicillin for almost over a decade, syphilis continues to be a disease of concern, and therefore the optimal management of syphilis continues to be a controversial topic [12, 40]. Presently, clinical guidelines suggest similar treatment regimens for various stages of syphilis. Penicillin continues to be the drug of choice to treat all stages of syphilis in all populations with tetracycline and cephalosporin acceptable alternate agents for some stages in nonpregnant person [12, 40]. Penicillin G remains the most suitable drug for treating infected persons with syphilis in all the stages. The stage and clinical manifestations of syphilis dictate the preparation used (i.e., benzathine, aqueous procaine, or aqueous crystalline), dosage, and length of treatment [11]. Persons with latent syphilis of unknown duration require longer treatment duration to ensure that those who did not acquire syphilis within the preceding period are adequately treated [11]. Novel and effective prevention strategies are keenly required to combat syphilis infection because currently there is no vaccine to prevent infection with syphilis [40]. Syphilis is a preventable and potentially eradicable disease [18]. Transmission is prevented, and subsequent new infections are also blocked when syphilis is treated, and hence it reduces the prevalence of syphilis in a population to a minimal level. Mathematical models play a very vital role in the study of the dynamic of infectious diseases; for example, [15] developed and analyzed a mathematical model that includes the basic stages of the disease and assumed that infected individuals acquire temporary immunity only after recovery from the latent and tertiary infections. [23] considered a SIR mathematical model to study the effect of presence of partial immunity and vaccine against syphilis infection in a population. The results reveal that health education leading to enhanced biological and behavioral protection against infection and the development of effective vaccine is the most effective way to control syphilis transmission in a high-risk population. [17] presented a new multistage deterministic model for the transmission dynamics of syphilis to qualitatively assess the role of loss of transitory immunity in the transmission process. They show that loss of transitory (natural) immunity can induce the phenomenon of backward bifurcation. [37] proposed a model for the transmission of syphilis in an MSM population that includes infection stages and treatment. [29] used a nonlinear mathematical model to study the dynamics of the spread of syphilis in heterogeneous settings with complications, and two stages of (primary and secondary stages) infection only were considered. [2] formulated a compartmental model to investigate the dynamics of the spread of syphilis in a sexually active population with some measure of disease control. The model undergoes the phenomenon of backward bifurcation and proposed that effective treatment strategies of syphilis in its primary and secondary infected individuals will help reduce the cases. In [26] an investigation was carried out to determine the synergistic interaction between HIV and syphilis using a mathematical model. The paper assessed the impact of syphilis treatment on the dynamics of syphilis and HIV coinfection in a human population where HIV treatment is not readily available or accessible to HIV infected individuals, and they proposed that if a concerted effort is exerted in the treatment of primary and secondary syphilis (in both singly and dually infected individuals), especially with high treatment rates for primary syphilis, then it will result in a reduction in the incidence of HIV (and its coinfection with syphilis) in the population.

However, until now, very few studies were conducted to investigate the optimal control strategies with the view to come up with best way of curtailing the spread of syphilis within a population. [38] incorporated in an epidemiological model for the transmission dynamics of syphilis a control variable to assess the effects of resistance strategies against the disease. He emphasizes the need to reevaluate the current control programs; the development of an effective vaccine associated with health education could be the best way to control syphilis in high-risk populations. [1] formulated and analyzed the dynamics of a syphilis model with the introduction of two controls, and the two time-independent controls represent strategies for improvement of the treatment and cure of the syphilis disease.

[34] used an epidemiological SEIR (Susceptible, Exposed, Infectious, Removed) type model for rubella epidemic via classical and fractional-order Caputo differential operators assuming the periodic transmission rate $\beta (t)$. [35] investigated the dynamics of measles infection with the help of mathematical operators called conformable derivatives of order *α* (the local derivative index) in the sense of Liouville–Caputo operator of order *β* (the iterated or fractionalizing index). An epidemiological model related with diarrhea transmission dynamics that occurred in Ghana during 2008–2018 was investigated by [36]. The epidemiological model was designed for the very first time with newly devised fractional Caputo-type operator having the fractional order *α* and the fractal dimension *τ*. [25] proposed a fractional-order epidemic model with two different operators called the classical Caputo operator and the Atangana–Baleanu–Caputo operator for the transmission of Covid-19 epidemic. The reproduction number $\mathcal{R}_{0}$ was obtained for the prediction and persistence of the disease in a population. [5] presented face masks simple but powerful weapons to protect individuals against Covid-19 spread. The focus of their research was to depict the transport of Covid-19 spread through wind with high speed. The stability of nonmonotone critical waves by antiweighted method for a kind of nonmonotone time-delayed reaction–diffusion equations, including Nicholson’s blowflies equation, which describes the population dynamics of a single species with age structure, was studied in [45]. In [6], homotopy transform methods, namely, homotopy analysis transform method and homotopy perturbation Sumudu transform method, were implemented to examine the fractional model for HIV infection of $CD4^{+}T$ lymphocyte cells. A mathematical model for the spread of Covid-19 was analyzed in [3] using theory of stability and optimal control. The proposed model was extended to the concept of nonlocal operators, in which the positiveness of the system solutions were established. [4] presented a detailed analysis of an important class of differential equations called stochastic equations with the new classes of differential operators with global derivatives of integer and noninteger orders. In an attempt to show the applicability of the operators, three epidemiological problems, namely, Zombie virus spread model, the Zika virus spread model, and Ebola model, were studied, and their results showed that more complex real-world problems could be depicted using the classes of differential equations studied.

In this research, we complement and extend the work of [29] by taking into cognizance the standard incidence rate and three stages of infection, namely, primary, secondary, and latent stages of infection of the disease transmission against two stages (primary and secondary) considered by [29], and according to recent revelations of various studies conducted by different authors, the early latent stage can also transmit the disease. However, we have also incorporated an optimal control by using three control strategies consisting of prevention and treatment to investigate the best control strategy that would assist in curtailing the spread of syphilis in a population based on the following assumptions: The latent stage of infection can also transmit the disease, individuals recovered from syphilis infection can also contact the disease after the loss of immunity, the population of both male and female are assumed to be sexually active in all stages of infection, individuals with syphilis in the primary stage can also transmit the disease, and individuals in the susceptible population can be infected with syphilis after having contact with either those in the primary, secondary, or individual at the latent stages of infection.

The paper is presented sectionwise as follows: The syphilis model is formulated in Sect. 2. Mathematical analysis of the syphilis model is presented in Sect. 3. The optimal control problem and analysis of the control problem are presented in Sect. 4. Numerical results and discussion are provided in Sect. 5. Finally, we conclude in Sect. 6.

## Formulation of syphilis model

The total population at time *t* represented as $N(t)$ is subdivided into smaller classes: susceptible males $S_{m}(t)$, susceptible females $S_{f}(t)$, males with primary stage syphilis infection $I_{mp}(t)$, females with primary stage syphilis infection $I_{fp}(t)$, males with secondary stage syphilis infection $I_{ms}(t)$, females with secondary stage syphilis infection $I_{fs}(t)$, males with latent stage syphilis infection $L_{m}(t)$, females with latent stage syphilis infection $L_{f}(t)$, recovered males $R_{m}(t)$, and recovered females $R_{f}(t)$. So

1 $$ N(t)= S_{m}(t)+S_{f}(t)+I_{mp}(t)+I_{fp}(t)+I_{ms}(t)+I_{fs}(t)+L_{m}(t)+L_{f}(t)+R_{m}(t)+R_{f}(t). $$

Recruitment into the susceptible males at time *t*, $S_{m}(t)$ is done by the increase in the number of sexually active individuals, who do not previously contact syphilis at rate $\pi _{m}$. The population is also increased by rate $\varphi _{m}$ of males who recovered from syphilis infection after the loss of immunity. The population of susceptible males reduces due to the development of newly infected males with syphilis who progress to the males with primary stage syphilis by a function $\alpha _{f}\psi (\frac{I_{fp}+I_{fs}+L_{f}}{N} )S_{m}$, where $\alpha _{f}$ represents the transmission probability of syphilis females, and *ψ* is the average number of sexual partners per unit time. The susceptible males reduce as a result of natural driven death at rate *μ*. So the equation becomes

$$ \frac{dS_{m}}{dt}=\pi _{m}+\varphi _{m}{R_{m}}- \alpha _{f}\psi \biggl( \frac{I_{fp}+I_{fs}+L_{f}}{N} \biggr)S_{m}-\mu {S_{m}}. $$

Recruitment into the susceptible females at time *t*, $S_{f}(t)$, is due to an increase in the number of sexually active females who did not previously contact syphilis at rate $\pi _{f}$. Recovery from syphilis infection and later loss of immunity also contribute to increasing in susceptible females at rate $\varphi _{m}$. This population is reduced by acquiring syphilis infection at the quantity $\alpha _{m}\psi (\frac{I_{mp}+I_{ms}+L_{m}}{N} )S_{f}$ and the migration of such a population to the females with syphilis at the primary stage of infection, where $\alpha _{m}$ is the transmission probability of syphilis infected male. The population is also reduced by death due to the natural factor at rate *μ*. Thus

$$ \frac{dS_{f}}{dt}=\pi _{f}+\varphi _{f}{R_{f}}- \alpha _{m}\psi \biggl( \frac{I_{mp}+I_{ms}+L_{m}}{N} \biggr)S_{f}-\mu {S_{f}}. $$

The population of males with primary stage syphilis at time *t*, $I_{mp}(t)$, increases due to progression of newly infected syphilis individuals from the susceptible male at the quantity $\alpha _{f}\psi (\frac{I_{fp}+I_{fs}+L_{f}}{N} )S_{m}$ and is reduced due to movement to males with secondary stage syphilis $I_{ms}$ at rate $\gamma _{m}$. This population is further decreased due to natural death at rate *μ* and presence of treatment using antibiotics at rate $\sigma _{m_{1}}$, so that

$$ \frac{dI_{mp}}{dt}=\alpha _{f}\psi \biggl( \frac{I_{fp}+I_{fs}+L_{f}}{N} \biggr)S_{m}-\gamma _{m}{I_{mp}}-\mu {I_{mp}}- \sigma _{m_{1}}{I_{mp}}. $$

The population of females with primary stage syphilis at time *t*, $I_{fp}(t)$, increases as a result of progression of newly infected females with syphilis infection from susceptible female at the quantity $\alpha _{m}\psi (\frac{I_{mp}+I_{ms}+L_{m}}{N} )S_{f}$ and is reduced due to progression to infected females with secondary stage syphilis $(I_{fs})$ at rate $\gamma _{f}$. There is a decrease in the population due to natural death at rate *μ* and treatment using antibiotics at rate $\rho _{f_{1}}$. Thus

$$ \frac{dI_{fp}}{dt}=\alpha _{m}\psi \biggl( \frac{I_{mp}+I_{ms}+L_{m}}{N} \biggr)S_{f}-\gamma _{f}{I_{f}p}-\mu {I_{f}p}- \rho _{f_{1}}I_{fp}. $$

The infected males with secondary stage syphilis infection at time *t*, $I_{ms}(t)$, increase due to progression of males with primary stage syphilis $(I_{mp})$ to males with secondary stage syphilis $(I_{ms})$ at rate $\gamma _{m}$. The population is reduced by the progression of males with secondary stage syphilis $(I_{ms})$ at rate $\beta _{m}$ to males with latent stage syphilis $(L_{m})$. The population is further reduced by death from natural factor at rate *μ* and due to the presence of treatment (antibiotics) at rate $\sigma _{m_{2}}$, so that the equation is given by

$$ \frac{dI_{ms}}{dt}=\gamma _{m}{I_{m}p}-\beta _{m}{I_{ms}}-\sigma _{m_{2}}{I_{ms}}- \mu {I_{ms}}. $$

Females with secondary stage syphilis population of at time *t*, $I_{fs}(t)$, increase due to progression of females with primary stage syphilis $(I_{fp})$ to females with secondary stage syphilis $(I_{fs})$ at rate $\gamma _{f}$. The population is reduced by the progression of females with secondary stage syphilis $(I_{fs})$ at rate $\beta _{f}$ to the latent stage syphilis $(L_{f})$ and is further reduced by a natural death at rate *μ* and due to treatment (antibiotics) at rate $\rho _{f2}$, so that the equation is given by

$$ \frac{dI_{fs}}{dt}=\gamma _{f}{I_{f}p}-\beta _{f}{I_{fs}}-\rho _{f_{2}}{I_{fs}}- \mu {I_{fs}}. $$

The population of males with latent syphilis at time *t*, $L_{m}(t)$, is increased by the progression of infected males with secondary stage syphilis $(I_{ms})$ at rate $\beta _{m}$ to the population of males with latent stage syphilis $(L_{m})$ and is reduced by a natural death at rate *μ*. This population is further reduced due to the presence of treatment (antibiotics) at rate $\sigma _{m_{3}}$, so that the equation is given by

$$ \frac{dL_{m}}{dt}=\beta _{m}{I_{ms}}-\mu {L_{m}}-\sigma _{m_{3}}{L_{m}}. $$

The population of females with latent stage syphilis at time *t*, $L_{f}(t)$, is increased by the progression of infected females with secondary stage syphilis $(I_{fs})$ at rate $\beta _{f}$ to the population of females with latent stage syphilis $(L_{f})$ and is reduced by natural death at rate *μ*. This population is further reduced due to the presence of treatment (antibiotics) at rate $\rho _{f_{3}}$, so that

$$ \frac{dL_{f}}{dt}=\beta _{f}{I_{fs}}-\mu {L_{f}}-\rho _{f_{3}}{L_{f}}. $$

The population of recovered males at time *t*, $R_{m}(t)$, is increased due to progression of treated males from primary, secondary, and latent stages of syphilis $(I_{mp}, I_{ms}, L_{m})$, respectively, at rates $\sigma _{m_{1}}$, $\sigma _{m_{2}}$, and $\sigma _{m_{3}}$, which are the treatment rates of syphilis infection in male population. This population is reduced due to natural death at rate *μ* and is further decreased due to loss of immunity acquired as a result of treatment and transfer of such individuals to susceptible male population at rate $\varphi _{m}$, so that

$$ \frac{dR_{m}}{dt}=\sigma _{m_{1}}I_{mp}+\sigma _{m_{2}}I_{ms}+\sigma _{m_{3}}L_{m}- \mu {R_{m}}-\varphi _{m}R_{m}. $$

The population of recovered females at time *t*, $R_{f}(t)$, is increased due to progression of treated females from primary, secondary, and latent stages of syphilis infections $(I_{fp}, I_{fs}, L_{f})$, respectively, at rates $\rho _{f_{1}}$, $\rho _{f_{2}}$, and $\rho _{f_{3}}$. This population is reduced due to natural death at rate *μ* and is further decreased due to loss of immunity acquired as a result of treatment and transfer of such an individual to the susceptible male population at rate $\varphi _{f}$:

$$ \frac{dR_{f}}{dt}=\rho _{f_{1}}I_{fp}+\rho _{f_{2}}I_{fs}+\rho _{f_{3}}L_{f}- \mu {R_{f}}-\varphi _{f}R_{f}. $$

**Figure 1:**
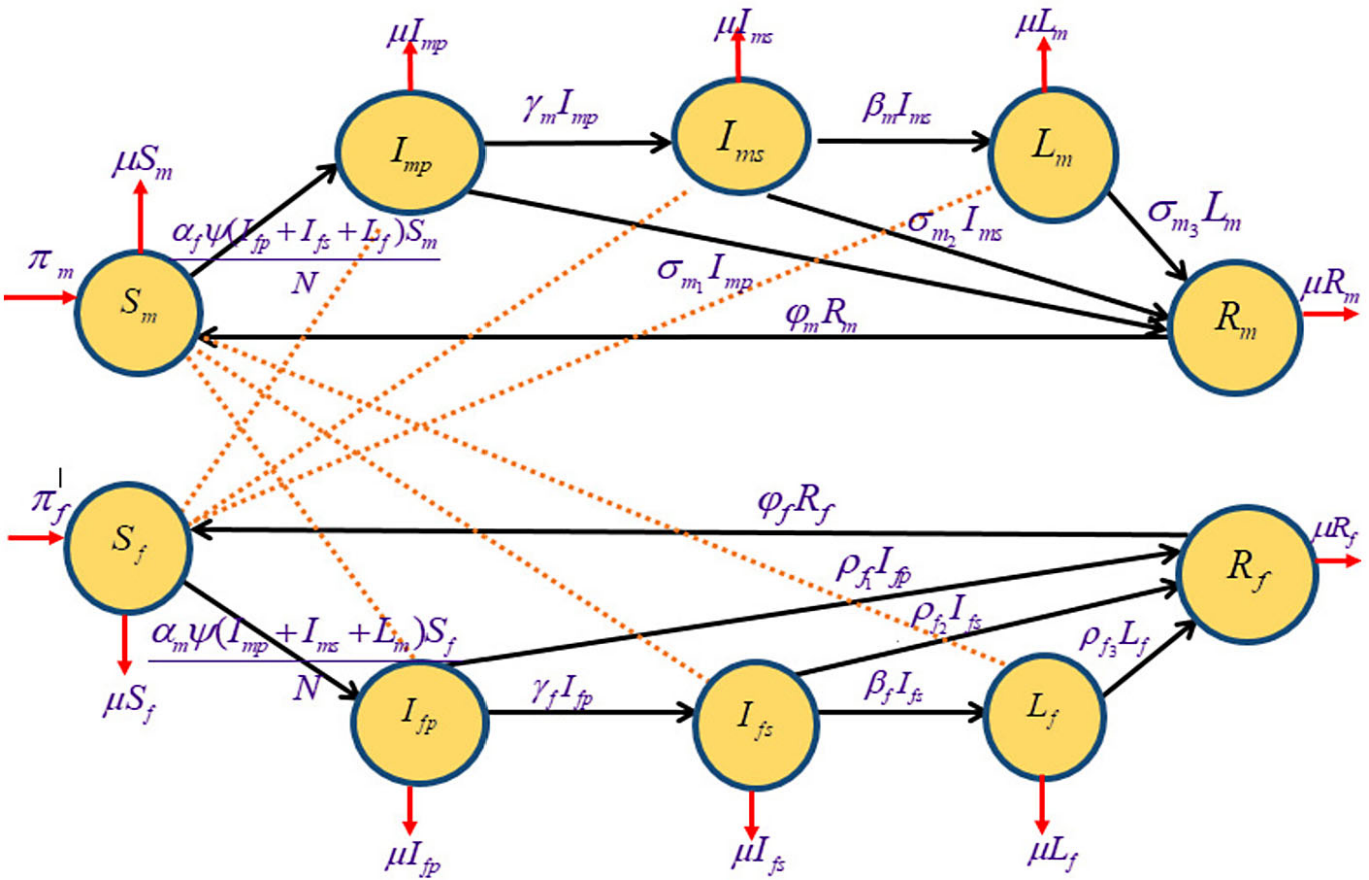
Model with three stages of infection and three control interventions

The model diagram is presented in Figure 1.

Therefore we present the syphilis model with three stages of infection:

2 $$ \begin{aligned} \textstyle\begin{cases} \frac{dS_{m}}{dt} =\pi _{m}+\varphi _{m}{R_{m}}-\alpha _{f}\psi (\frac{I_{fp}+I_{fs}+L_{f}}{N} )S_{m}-\mu {S_{m}}, \\ \frac{dI_{mp}}{dt} =\alpha _{f}\psi ( \frac{I_{fp}+I_{fs}+L_{f}}{N} )S_{m}-\gamma _{m}{I_{mp}}-\mu {I_{mp}}- \sigma _{m_{1}}{I_{mp}}, \\ \frac{dI_{ms}}{dt} =\gamma _{m}{I_{m}p}-\beta _{m}{I_{ms}}-\sigma _{m_{2}}{I_{ms}}- \mu {I_{ms}}, \\ \frac{dL_{m}}{dt} =\beta _{m}{I_{ms}}-\mu {L_{m}}-\sigma _{m_{3}}{L_{m}}, \\ {} \frac{dR_{m}}{dt} =\sigma _{m_{1}}I_{mp}+\sigma _{m_{2}}I_{ms}+ \sigma _{m_{3}}L_{m}-\mu {R_{m}}-\varphi _{m}R_{m}, \\ {} \frac{dS_{f}}{dt} =\pi _{f}+\varphi _{f}{R_{f}}-\alpha _{m}\psi (\frac{I_{mp}+I_{ms}+L_{m}}{N} )S_{f}-\mu {S_{f}}, \\ {} \frac{dI_{fp}}{dt} =\alpha _{m}\psi ( \frac{I_{mp}+I_{ms}+L_{m}}{N} )S_{f}-\gamma _{f}{I_{f}p}-\mu {I_{f}p}- \rho _{f_{1}}I_{fp}, \\ {} \frac{dI_{fs}}{dt} =\gamma _{f}{I_{f}p}-\beta _{f}{I_{fs}}-\rho _{f_{2}}{I_{fs}}- \mu {I_{fs}},{} \\ \frac{dL_{f}}{dt} =\beta _{f}{I_{fs}}-\mu {L_{f}}-\rho _{f_{3}}{L_{f}}, \\ {} \frac{dR_{f}}{dt} =\rho _{f_{1}}I_{fp}+\rho _{f_{2}}I_{fs}+\rho _{f_{3}}L_{f}- \mu {R_{f}}-\varphi _{f}R_{f}, \end{cases}\displaystyle \end{aligned} $$

with the initial conditions

3 $$ \textstyle\begin{cases} S_{m}(0)=S_{m0}(0)\geq 0,\qquad I_{mp}(0)=I_{mp0} \geq 0,\\ I_{ms}(0)=I_{ms0}(0) \geq 0,\qquad L_{m}(0)=L_{m0}(0) \geq 0, \\ S_{f}(0)=S_{f0}(0)\geq 0,\qquad I_{fp}(0)=I_{fp0} \geq 0,\\ I_{fs}(0)=I_{fs0}(0) \geq 0,\qquad L_{f}(0)=L_{f0}(0) \geq 0. \end{cases} $$

The associated subclasses are added to get the dynamics of the total population of system (2), which yields

4 $$ \frac{dN}{dt}=\pi _{m}+\pi _{f} -\mu N. $$

**Table 1:** Syphilis model variables

Variable	Description
$S_{m}(t)$	Susceptible males at time *t*
$I_{mp}(t)$	Males with primary stage syphilis at time *t*
$I_{ms}(t)$	Males with secondary stage syphilis at time *t*
$L_{m}(t)$	Males with latent stage syphilis at time *t*
$R_{m}(t)$	Recovered males from syphilis at time *t*
$S_{f}(t)$	Susceptible females at time *t*
$I_{fp}(t)$	Females with primary stage syphilis at time *t*
$I_{fs}(t)$	Females with secondary stage syphilis at time *t*
$L_{f}(t)$	Females with latent stage syphilis at time *t*
$R_{f}(t)$	Recovered females from syphilis at time *t*

### Basic properties

#### Theorem 1

*Let* $S_{m}(0),I_{mp}(0),I_{ms}(0),L_{m}(0),R_{m}(0),S_{f}(0),I_{fp}(0),I_{fs}(0),L_{f}(0),R_{f}(0)>0$ *be nonnegative initial conditions*. *Then system* (2) *has a nonnegative solution* $S_{m}(t),I_{mp}(t),I_{ms}(t), L_{m}(t), R_{m}(t),S_{f}(t),I_{fp}(t),I_{fs}(t),L_{f}(t),R_{f}(t)>0$ *for all* $t>0$. *Moreover*, $\limsup_{t\to \infty }N(t)\leq \frac{\pi _{m}+\pi _{f}}{\mu }$. *In addition*, *if* $N(0)\leq \frac{\pi _{m}+\pi _{f}}{\mu }$, *then* $N(t)\leq \frac{\pi _{m}+\pi _{f}}{\mu }$ *is the feasible region for system* (2)

5 $$ \Omega = \biggl\lbrace (S_{m},I_{mp},I_{ms},L_{m}, R_{m},S_{m},I_{fp},I_{fs}, L_{f}, R_{f})\in \mathbb{R}_{+}^{10}:N_{P} \leq \frac{\pi _{m}+\pi _{f}}{\mu } \biggr\rbrace $$

*is positively invariant and attracting with respect to system* (2).

#### Proof

From the first equation of system (2) we have

6 $$ \frac{dS_{m}}{dt}+ \frac{\alpha _{f}\psi (I_{fp}(t)+I_{fs}(t))+L_{f}(t))}{N(t)}S_{m}(t)+ \mu S_{m}(t)\geq 0. $$

From time $t=0$ to $t=t$, integrating (6), we get

$$ \frac{d}{dt} \biggl[S_{m}(t) \exp \biggl\lbrace \int _{0}^{t} \frac{\alpha _{f}\psi (I_{fp}(t)+I_{fs}(t)+L_{f}(t))}{N(t)}( \overline{\omega })\,d\overline{\omega } +\mu t \biggr\rbrace \biggr] \geq 0. $$

This means that

$$ S_{m}(t)\geq S_{m}(0) \exp \biggl\lbrace - \biggl( \int _{0}^{t} \frac{\alpha _{f}\psi (I_{fp}(t)+I_{fs}(t)+L_{f}(t))}{N(t)}( \overline{\omega })\,d\overline{\omega } +\mu t \biggr) \biggr\rbrace > 0,\quad \forall t > 0. $$

We applied a similar method to establish that $I_{mp}(t),I_{ms}(t),L_{m}(t),R_{m}(t),S_{f}(t), I_{fp}(t),I_{fs}(t), L_{f}(t),R_{f}(t)>0$ remain nonnegative for all $t>0$. Equation (4) is used to prove the second part of the theorem, which says that model system (2) is positively invariant, so that $N(t)\leq \frac{(\pi _{m}+\pi _{f})(1-\exp ^{-\mu t})}{\mu }+ \frac{\mu N(0)\exp ^{-\mu t}}{\mu } $. It follows that as $t\longrightarrow \infty $ $N(t)\leq \frac{\pi _{m}+\pi _{f}}{\mu }$. Furthermore, if $N(0)\leq \frac{\pi _{m}+\pi _{f}}{\mu }$, then $N(t)\leq \frac{\pi _{m}+\pi _{f}}{\mu }$. This establishes that Ω is the manifold on which the population has nonzero size.

This proves the boundedness of the solutions inside Ω. Hence the solutions to system (2) are positively invariant and attracting in a region Ω. Note that system (2) is feasible biologically and mathematically well posed in Ω from Theorem 1. □

## Analysis of the syphilis model

This section establishes the syphilis-free equilibrium state, syphilis-present equilibrium state, derived the basic reproduction number, and carryout stability analysis.

### Equilibrium points

The equilibrium points of system (2) are established by setting it to zero:

7 $$ \begin{aligned} \textstyle\begin{cases} \frac{dS_{m}}{dt} =\pi _{m}+\varphi _{m}{R_{m}}-\alpha _{f}\psi (\frac{I_{fp}+I_{fs}+L_{f}}{N} )S_{m}-\mu {S_{m}}=0, \\ {} \frac{dI_{mp}}{dt} =\alpha _{f}\psi ( \frac{I_{fp}+I_{fs}+L_{f}}{N} )S_{m}-\gamma _{m}{I_{mp}}-\mu {I_{mp}}- \sigma _{m_{1}}{I_{mp}}=0, \\ {} \frac{dI_{ms}}{dt} =\gamma _{m}{I_{m}p}-\beta _{m}{I_{ms}}-\sigma _{m_{2}}{I_{ms}}- \mu {I_{ms}}=0, \\ \frac{dL_{m}}{dt} =\beta _{m}{I_{ms}}-\mu {L_{m}}-\sigma _{m_{3}}{L_{m}}=0, \\ {} \frac{dR_{m}}{dt} =\sigma _{m_{1}}I_{mp}+\sigma _{m_{2}}I_{ms}+ \sigma _{m_{3}}L_{m}-\mu {R_{m}}-\varphi _{m}R_{m}=0, \\ {} \frac{dS_{f}}{dt} =\pi _{f}+\varphi _{f}{R_{f}}-\alpha _{m}\psi (\frac{I_{mp}+I_{ms}+L_{m}}{N} )S_{f}-\mu {S_{f}}=0, \\ {} \frac{dI_{fp}}{dt} =\alpha _{m}\psi ( \frac{I_{mp}+I_{ms}+L_{m}}{N} )S_{f}-\gamma _{f}{I_{f}p}-\mu {I_{f}p}- \rho _{f_{1}}I_{fp}=0, \\ {} \frac{dI_{fs}}{dt} =\gamma _{f}{I_{f}p}-\beta _{f}{I_{fs}}-\rho _{f_{2}}{I_{fs}}- \mu {I_{fs}}=0,{} \\ \frac{dL_{f}}{dt} =\beta _{f}{I_{fs}}-\mu {L_{f}}-\rho _{m_{3}}{L_{f}}, \\ {} \frac{dR_{f}}{dt} =\rho _{f_{1}}I_{fp}+\rho _{f_{2}}I_{fs}+\rho _{f_{3}}L_{f}- \mu {R_{f}}-\varphi _{f}R_{f}=0. \end{cases}\displaystyle \end{aligned} $$

When there is no syphilis infection, system (2) has a steady state, which is termed a syphilis-free equilibrium. To obtain the nature of stability of the syphilis-free equilibrium, we computed and evaluated the Jacobian of system (2) at syphilis-free equilibrium. The local stability of the syphilis-free equilibrium is determined using the signs of the eigenvalues of the Jacobian. The syphilis-free equilibrium for system (2) is

8 $$ \bigl({S_{m}^{0}},{I_{mp}^{0}},{I_{ms}^{0}},{L_{m}^{0}},{R_{m}^{0}},{S_{f}^{0}},{I_{fp}^{0}},{I_{fs}^{0}},{L_{f}^{0}},{R_{f}^{0}} \bigr)= \biggl(\frac{\pi _{m}}{\mu },0,0,0,0,\frac{\pi _{f}}{\mu },0,0,0,0 \biggr). $$

This simply means that in the absence of syphilis infection the susceptible males and females proportionally change with their recruitment rate to their death rate.

For the second equilibrium point, let $E^{**}=(S_{m}^{**},I_{mp}^{**},I_{ms}^{**},L_{m}^{**},R_{m}^{**},S_{f}^{**},I_{fp}^{**},I_{fs}^{**},L_{f}^{**},R_{f}^{**})$ be the syphilis-present equilibrium of model (2). At equilibrium state, let $\lambda _{m}^{**}= \frac{\alpha _{m}\psi (I_{mp}^{**}+I_{ms}^{**}+L_{m}^{**})}{N^{**}} $, $\lambda _{f}^{**}= \frac{\alpha _{f}\psi (I_{fp}^{**}+I_{fs}^{**}+L_{f}^{**})}{N^{**}} $ be the forces of infection, and let $N^{**}=S_{m}^{**}+I_{mp}^{**}+I_{ms}^{**}+L_{m}^{**}+R_{m}^{**}+S_{f}^{**}+I_{fp}^{**}+I_{fs}^{**}+L_{f}^{**}+R_{f}^{**}$. Then solving system (2) at steady state yields

9 $$ \begin{aligned} \textstyle\begin{cases} {S_{m}^{**}}= \frac{k_{1}k_{2}I_{ms}^{\ast }}{\gamma _{m}({\lambda _{f}^{**}+\mu })}, \\ {I_{mp}^{**}}= \frac{k_{2}I_{ms}^{\ast }}{\gamma _{m}}, \\ {I_{ms}^{**}}= \frac{\pi _{m} \gamma _{m} k_{3}k_{4}}{k_{1}k_{2}k_{3}k_{4}-(\sigma _{m_{3}} \beta _{m}\gamma _{m} \varphi _{m}+k_{3}\sigma _{m_{2}}\gamma _{m}\psi _{m}+\varphi _{m} \sigma _{m_{1}}k_{2}k_{3})}, \\ {L_{m}^{**}}= \frac{\beta _{m} I_{ms}^{\ast }}{k_{3}}, \\ {R_{m}^{**}}= \frac{k_{1}k_{2}I_{ms}^{**}-\gamma _{m}\pi _{m}}{\gamma _{m} \varphi _{m}}, \\ {S_{f}^{**}}= \frac{h_{1}h_{2}I_{fs}^{**}}{\gamma _{f}({\lambda _{m}^{\ast }+\mu })}, \\ {I_{fp}^{**}}= \frac{h_{2}I_{fs}^{\ast }}{\gamma _{f}}, \\ {I_{fs}^{**}}= \frac{\pi _{f} \gamma _{f} h_{3}h_{4}}{h_{1}h_{2}h_{3}h_{4}-(\rho _{f_{3}} \beta _{f}\gamma _{f} \varphi _{f}+h_{3}\rho _{f_{2}}\gamma _{f}\psi _{f}+\varphi _{f} \rho _{f_{1}}h_{2}h_{3})}, \\ {L_{f}^{**}}= \frac{\beta _{f} I_{fs}^{**}}{h_{3}}, \\ {R_{f}^{**}}= \frac{(\gamma _{f}+\mu +\rho _{f_{1}})(\beta _{f}+{\rho _{f_{2}}+\mu })I_{fs}^{**}-\gamma _{f} \pi _{f}}{\gamma _{f} \varphi _{2}}, \end{cases}\displaystyle \end{aligned} $$

where $k_{1}=\gamma _{m}+\mu +\sigma _{m_{1}}$, $k_{2}=\beta _{m}+\sigma _{m_{2}}+ \mu $, $k_{3}=\mu +\sigma _{m_{3}}$, $k_{4}=\mu +\varphi _{m}$, and $h_{1}=\gamma _{f}+\mu +\rho _{f_{1}}$, $h_{2}=\beta _{f}+\rho _{f_{2}}+ \mu $, $h_{3}=\mu +\rho _{f_{3}}$, $h_{4}=\mu +\varphi _{f} $.

### Basic reproduction number

The average number of secondary infections caused by one infectious person when the entire population is susceptible is termed the basic reproduction number $\mathcal{R}_{0}$. The epidemiological threshold of syphilis is denoted by $\mathcal{R}_{0}=\rho (F{V}^{-1})$, where *ρ* is the dominant eigenvalue. To find the basic reproduction number of system (2), we adopted the techniques in [41] to get

$$ F= \begin{pmatrix} \alpha _{f}\psi (\frac{I_{fp}+I_{fs}+L_{f}}{N} )S_{m} \\ 0 \\ 0 \\ \alpha _{m}\psi (\frac{I_{mp}+I_{ms}+L_{m}}{N} )S_{f} \\ 0 \\ 0 \end{pmatrix} \quad \mbox{and}\quad V= \begin{pmatrix} (\gamma _{m}+\mu +\sigma _{m_{1}})I_{mp} \\ -\gamma _{m} I_{mp}+(\beta _{m}+\mu +\sigma _{m_{2}})I_{ms} \\ -\beta _{m} I_{ms}+(\mu +\sigma _{m_{3}})L_{m} \\ (\gamma _{f}+\mu +\rho _{f_{1}})I_{fp} \\ -\gamma _{f} I_{fp}+(\beta _{f}+\mu +\rho _{f_{2}})I_{fs} \\ -\beta _{f} I_{fs}+(\mu +\rho _{f_{3}})L_{f} \end{pmatrix} . $$

The matrices *F* and *V* contain new infection terms and transition terms, respectively, in system (2). Evaluating the Jacobian matrices of *F* and *V* at syphilis-free equilibrium yields

$$ F= \begin{pmatrix} 0&0&0&\frac{\alpha _{f}\psi \pi _{m}}{\pi _{m}+\pi _{f}}&\frac{\alpha _{f}\psi \pi _{m}}{\pi _{m}+\pi _{f}}&\frac{\alpha _{f}\psi \pi _{m}}{\pi _{m}+\pi _{f}} \\ 0&0&0&0&0&0 \\ 0&0&0&0&0&0 \\ \frac{\alpha _{m}\psi \pi _{f}}{\pi _{m}+\pi _{f}}&\frac{\alpha _{m}\psi \pi _{f}}{\pi _{m}+\pi _{f}}&\frac{\alpha _{m}\psi \pi _{f}}{\pi _{m}+\pi _{f}}&0&0&0 \\ 0&0&0&0&0&0 \\ 0&0&0&0&0&0 \end{pmatrix} $$

and

$$ V= \begin{pmatrix} \gamma _{m}+\mu +\sigma _{m_{1}}&0&0&0&0&0 \\ -\gamma _{m}&\beta _{m}+\mu +\sigma _{m_{2}}&0&0&0&0 \\ 0&-\beta _{m}&\mu +\sigma _{m_{3}}&0&0&0 \\ 0&0&0&\gamma _{f}+\mu +\rho _{f_{1}}&0&0 \\ 0&0&0&-\gamma _{f}&\beta _{f}+\mu +\rho _{f_{2}}&0 \\ 0&0&0&0&-\beta _{f}&\mu +\rho _{f_{3}} \end{pmatrix} . $$

Therefore system (2) has the basic reproduction number

10 $$ \mathcal{R}_{0}=\sqrt{ \frac{\psi ^{2}\alpha _{f}\alpha _{m}\pi _{m}\pi _{f}(\beta _{m} \gamma _{m}+\gamma _{m} q_{2}+q_{2} q_{3})(\beta _{f} \gamma _{f}+\gamma _{f} q_{6}+q_{5} q_{6})}{(\pi _{m}+\pi _{f})^{2}q_{1} q_{2} q_{3} q_{4} q_{5} q_{6}}}, $$

where $q_{1}=(\gamma _{m}+\mu +\sigma _{m_{1}})$, $q_{2}=(\beta _{m}+\mu +\sigma _{m_{2}})$ $q_{3}=(\mu +\sigma _{m_{3}})$, $q_{4}=(\gamma _{f}+\mu +\rho _{f_{1}})$, $q_{5}=(\beta _{f}+\mu +\rho _{f_{2}})$, and $q_{6}=(\mu +\rho _{f_{3}})$.

### Local stability of the syphilis-free equilibrium

#### Theorem 2

*The syphilis*-*free equilibrium of system* (2) *is locally asymptotically stable if* $\mathcal{R}_{0}<1$ *and unstable otherwise*.

#### Proof

The Jacobian matrix of system (2) at syphilis-free equilibrium is

$$ J\bigl(E^{0}\bigr)= \begin{pmatrix} -r_{1} & 0 & 0 & 0 &\varphi _{1} & 0 & - \frac{\alpha _{f} \psi \pi _{m}}{\pi _{m}+\pi _{f}}& - \frac{\alpha _{f}\psi \pi _{m}}{\pi _{m}+\pi _{f}} & - \frac{\alpha _{f}\psi \pi _{m}}{\pi _{m}+\pi _{f}} & 0 \\ 0 & -r_{2} & 0 & 0 & 0 & 0 & \frac{\alpha _{f} \psi \pi _{m}}{\pi _{m}+\pi _{f}}& \frac{\alpha _{f}\psi \pi _{m}}{\pi _{m}+\pi _{f}}& \frac{\alpha _{f}\psi \pi _{m}}{\pi _{m}+\pi _{f}}& 0 \\ 0& \gamma _{m} & -r_{3} & 0 & 0 & 0 & 0 & 0 & 0 & 0 \\ 0& 0& \beta _{m} & -r_{4} & 0 & 0 & 0 & 0 & 0 & 0 \\ 0& \sigma _{m_{1}} & \sigma _{m_{2}} & \sigma _{m_{3}} & -r_{5} & 0 & 0 & 0 & 0 & 0 \\ 0& -\frac{\alpha _{f}\psi \pi _{f}}{\pi _{m}+\pi _{f}} & - \frac{\alpha _{f}\psi \pi _{f}}{\pi _{m}+\pi _{f}} & - \frac{\alpha _{f}\psi \pi _{f}}{\pi _{m}+\pi _{f}} & 0 & -r_{6} & 0 & 0 & 0 & \varphi _{f} \\ 0&\frac{\alpha _{m}\psi \pi _{f}}{\pi _{m}+\pi _{f}} & \frac{\alpha _{m}\psi \pi _{f}}{\pi _{m}+\pi _{f}} & \frac{\alpha _{m}\psi \pi _{f}}{\pi _{m}+\pi _{f}} & 0 & 0 & -r_{7} & 0 & 0 & 0 \\ 0& 0 & 0 & 0 & 0 & 0 & \gamma _{f} & -r_{8} & 0 & 0 \\ 0& 0 & 0 & 0 & 0 & 0 & 0 & \beta _{f} & -r_{9} & 0 \\ 0& 0 & 0 & 0 & 0 & 0 & \rho _{f_{1}} & \rho _{f_{2}} & \rho _{f_{3}} & -r_{10} \end{pmatrix} , $$

where $r_{1}=\mu $, $r_{2}=(\gamma _{m}+\mu +\sigma _{m_{1}})$, $r_{3}=(\beta _{m}+\mu +\sigma _{m_{2}})$, $r_{4}=(\mu +\sigma _{m_{3}})$, $r_{5}=(\mu +\varphi _{m})$, $r_{6}=\mu $, $r_{7}=(\gamma _{f}+\mu +\rho _{f_{1}})$, $r_{8}=(\gamma _{f}+\mu +\rho _{f_{2}})$, $r_{9}=(\mu +\rho _{f_{3}})$, and $r_{10}=(\mu +\varphi _{f})$.

From the matrix $J(E^{0})$ we have $\lambda _{1}=-\mu <0$, $\lambda _{2}=-(\mu +\varphi _{m})< 0$, $\lambda _{3}=- \mu< 0$, and $\lambda _{4}=-(\mu +\varphi _{f})< 0$, so that the matrix $J(E^{0})$ reduces to

11 $$ \begin{pmatrix} -r_{2} & 0 & 0 & \frac{\alpha _{f} \psi \pi _{m}}{\pi _{m}+\pi _{f}}&\frac{\alpha _{f} \psi \pi _{m}}{\pi _{m}+\pi _{f}}&\frac{\alpha _{f} \psi \pi _{m}}{\pi _{m}+\pi _{f}} \\ \gamma _{m} & -r_{3} & 0 & 0&0&0 \\ 0 & \beta _{m} & -r_{4} & 0&0&0 \\ \frac{\alpha _{m}\psi \pi _{f}}{\pi _{m}+\pi _{f}} & \frac{\alpha _{m}\psi \pi _{f}}{\pi _{m}+\pi _{f}} & \frac{\alpha _{m}\psi \pi _{f}}{\pi _{m}+\pi _{f}} & -r_{7}&0&0 \\ 0 & 0 & 0 & \gamma _{f}&-r_{8}&0 \\ 0 & 0 & 0 & 0&\beta _{f}&-r_{9} \end{pmatrix} . $$

The characteristic equation for (11) is hereby defined as follows:

12 $$ \lambda ^{6}+D_{1} \lambda ^{5}+D_{2} \lambda ^{4}+D_{3} \lambda ^{3}+D_{4} \lambda ^{2}+D_{5} \lambda + D_{6}=0, $$

where the coefficients of (12) are given as $D_{1}=(r_{2}+r_{3}+r_{4}+r_{7}+r_{8}+r_{9})$, $D_{2}=r_{9}(r_{2}+r_{3}+r_{4}+r_{7}+r_{8})+r_{3}(r_{2}+r_{3})+r_{2}r_{3}+r_{7}(r_{2}+r_{3}+r_{4})+r_{8}(r_{2}+r_{3}+r_{4}+r_{7})-b_{1}c_{1}$, $D_{3}=(r_{9}(r_{4}(r_{2}+r_{3})+r_{2}r_{3}+r_{7}(r_{2}+r_{3}+r_{4})+r_{8}(r_{2}+r_{3}+r_{4}+r_{7}))+r_{7}(r_{4}(r_{2}+r_{3})+r_{2}r_{3}) +r_{8}(r_{4}(r_{2}+r_{3})+r_{2}r_{3}+r_{7}(r_{2}+r_{3}+r_{4}))+b_{1}c_{1}r_{2}+r_{2}r_{3}r_{4}-b_{1}c_{1}(2+ \gamma _{f}+\gamma _{m})+(r_{2}+r_{3}+r_{4}))$, $D_{4}=(c_{1}(\gamma _{m}b_{1}r_{2}+\gamma _{m}b_{1}r_{3})+r_{8}(r_{7}(r_{4}(r_{2}+r_{3})+r_{2}r_{3})- \gamma _{m}b_{1}c_{1}+b_{1}c_{1}r_{2}+r_{2}r_{3}r_{4}-b_{1}c_{1}(r_{2}+r_{3}+r_{4}))-( \gamma _{m}b_{1}c_{1}-b_{1}c_{1}r-2)(r_{2}+r_{3}+r_{4})+\gamma _{f}(b_{1}c_{1}r_{2}- \gamma _{m}b_{1}c_{1}+b_{1}c_{1}r_{7})+r_{9}(r_{7}(r_{4}(r_{2}+r_{3})+r_{2}r_{3})+r_{8}(r_{4}(r_{2}+r_{3})-b_{1}c_{1}+r_{2}r_{3}+r_{7}(r_{2}+r_{3}+r_{4}))- \gamma _{f}b_{1}c_{1}-\gamma _{m}b_{1}c_{1}+b_{1}c_{1}r_{2}+r_{2}r_{3}r_{4}-b_{1}c_{1}(r_{2}+r_{3}+r_{4}))-b_{1}c_{1}r_{2}^{2}-b_{1}c_{1}(r_{4}(r_{2}+r_{3})+r_{2}r_{3})+r_{2}r_{3}r_{4}r_{7}- \gamma _{f}b_{1}c_{1}(r_{2}+r_{3}+r_{4}+r_{7})-\beta _{f}\gamma _{f}b_{1}c_{1}- \beta _{m}\gamma _{m}b_{1}c_{1})$, $D_{5}=(\beta _{f}(\gamma _{f}(b_{1}c_{1}r_{2}-\gamma _{m}b_{1}c_{1}+b_{1}c_{1}r_{7})+ \gamma _{f}b_{1}c_{1}r_{8})-\gamma _{f}(r_{7}(b_{1}c_{1}r_{2}-\gamma _{m}b_{1}c_{1}+b_{1}c_{1}r_{7})-c_{1}( \gamma _{m}b_{1}r_{2}+\gamma _{m}b_{1}r_{3})+c_{1}(c_{1}b_{1}^{2}+b_{1}r_{2}^{2})+ \beta _{m}\gamma _{m}b_{1}c-1)-r_{9}((\gamma _{m}b_{1}c_{1}-b_{1}c_{1}r_{2})(r_{2}+r_{3}+r_{4})-r_{8}(r_{7}(r_{4}(r_{2}+r_{3})+r_{2}r_{3})- \gamma _{m}b_{1}c_{1}+b_{1}c_{1}r_{2}+r_{2}r_{3}r_{4}-b_{1}c_{1}(r_{2}+r_{3}+r_{4}))-c_{1}( \gamma _{m}b_{1}r_{2}+\gamma _{m}b_{1}r_{3})-\gamma _{f}(b_{1}c_{1}r_{2}- \gamma _{m}b_{1}c_{1}+b_{1}c_{1}r_{7})+b_{1}c_{1}r_{2}^{2}+b_{1}c_{1}(r_{4}(r_{2}+r_{3})+r_{2}r-3)-r_{2}r_{3}r_{4}r_{7}+ \gamma _{f}b_{1}c-1(r_{2}+r_{3}+r_{4}+r_{7})+\beta _{m}\gamma _{m}b_{1}c-1)-r_{8}(( \gamma _{m}b_{1}c_{1}-b_{1}c_{1}r_{2})(r_{2}+r_{3}+r_{4})-c_{1}( \gamma _{m}b_{1}r_{2}+\gamma _{m}b_{1}r_{3})+b_{1}c_{1}r_{2}^{2}+b_{1}c_{1}(r_{4}(r_{2}+r_{3})+r_{2}r_{3})-r_{2}r-3r-4r_{7}+ \beta _{m}\gamma _{m}b_{1}c_{1})+\gamma _{f}(b_{1}c_{1}r_{2}-\gamma _{m}b_{1}c_{1}+b_{1}c_{1}r_{7})(r_{2}+r_{3}+r_{4}+r_{7})- \gamma _{f}b_{1}c_{1}(r_{4}(r_{2}+r_{3})-b_{1}c_{1}+r_{2}r_{3}+r_{7}(r_{2}+r_{3}+r_{4}))- \beta _{f}\gamma _{f}b_{1}c_{1}(r_{2}+r_{3}+r_{4}+r_{7}+r_{8}))$, $D_{6}=(\beta _{f}(\gamma _{f}(b_{1}c_{1}r_{2}-\gamma _{m}b_{1}c_{1}+b_{1}c_{1}r_{7})+ \gamma _{f}b_{1}c_{1}r_{8})(r_{2}+r_{3}+r_{4}+r_{7}+r_{8})-r_{9}( \gamma _{f}(r_{7}(b_{1}c_{1}r_{2}-\gamma _{m}b_{1}c_{1}+b_{1}c_{1}r_{7})-c_{1}( \gamma _{m}b_{1}r_{2}+\gamma _{m}b_{1}r_{3})+c_{1}(c_{1}b_{1}^{2}+b_{1}r_{2}^{2})+ \beta _{m}\gamma _{m}b_{1}c_{1})+r_{8}((\gamma _{m}b_{1}c_{1}-b_{1}c_{1}r_{2})(r_{2}+r_{3}+r_{4})-c_{1}( \gamma _{m}b_{1}r_{2}+\gamma _{m}b_{1}r_{3})+b_{1}c_{1}r_{2}^{2}+b_{1}c_{1}(r_{4}(r_{2}+r_{3})+r_{2}r_{3})-r_{2}r_{3}r_{4}r_{7}+ \beta _{m}\gamma _{m}b_{1}c_{1})-\gamma _{f}(b_{1}c_{1}r_{2}-\gamma _{m}b_{1}c_{1}+b_{1}c_{1}r_{7})(r_{2}+r_{3}+r_{4}+r_{7})+ \gamma _{f}b_{1}c_{1}(r_{4}(r_{2}+r_{3})-b_{1}c_{1}+r_{2}r_{3}+r_{4}(r_{2}+r_{3}+r_{4})))- \beta _{f}(\gamma _{f}(r_{7}(b_{1}c_{1}r_{2}-\gamma _{m}b_{1}c_{1}+b_{1}c_{1}r_{7})-c_{1}( \gamma _{m}b_{1}r_{2}+\gamma _{m}b_{1}r_{1})+c_{1}(c_{1}b_{1}^{2}+b_{1}r_{2}^{2})+ \beta _{m}\gamma _{m}b_{1}c-1)+r_{8}(\gamma _{f}(b_{1}c_{1}r_{2}- \gamma _{m}b_{1}c_{1}+b_{1}c_{1}r_{7})+\gamma _{f}b_{1}c_{1}r_{8}))- \beta _{f}\gamma _{f}b_{1}c_{1}(r_{4}(r_{2}+r_{3})-b_{1}c_{1}+r_{2}r_{3}+r_{7}(r_{2}+r_{3}+r_{4})+r_{8}(r_{2}+r_{3}+r_{4}+r_{7}))) $.

By applying the Routh–Hurwitz criterion (which states that all roots of the polynomial equation (12) have a negative real part if and only if the coefficients are positive and the determinant of the matrices $H_{i}>1$ for $i=1,\ldots,6$) it is clear that $D_{1}>0$. Therefore, if $D_{j}>0$ for $j=2,\ldots,6$ and the necessary condition for the Routh–Hurwitz criterion for the sixth-order characteristic polynomial in (12) is satisfied, then we conclude that the syphilis-free equilibrium is locally asymptotically stable (LAS). □

### Syphilis global asymptotic stability

The approach in [8] is adopted to investigate the global asymptotic stability (GAS) of the syphilis-free equilibrium for the model (2).

#### Lemma 1

*Let system* (2) *be of the form*

13 $$ \begin{aligned} \textstyle\begin{cases} \frac{dQ}{dt}=F(Q,Z), \\ \frac{dZ}{dt}=G(Q,Z),\quad (Q,0)=0, \end{cases}\displaystyle \end{aligned} $$

*where* $Q=(S_{m}, R_{m}, S_{f}, R_{f})$ *and* $Z=(I_{mp}, I_{ms}, L_{m}, I_{fp}, I_{fs}, L_{m})$, *and the components of* $Q\in \mathbb{R}^{4}$ *represent the population that is not infected*, *and the components of* $Z\in \mathbb{R}^{6}$ *represent the infected population* [8]. *Consider the syphilis*-*free equilibrium* $E^{0}=(Q^{0},0)$, *where*

14 $$ Q^{0}= \biggl(\frac{\pi _{m}}{\mu },0,0,0,0,\frac{\pi _{f}}{\mu },0,0,0,0 \biggr). $$

*The conditions that must be met to guarantee the global asymptotic stability are*: $H_{1}:\frac{dQ}{dt}=F(Q^{0},0)$, *where* $Q^{0}$ *is* (*GAS*). $H_{2}:G(Q,Z)=PZ-\hat{G}(Q,Z)$, $\hat{G}(Q,Z)\geq 0$ *for* $(Q,Z)\in \Omega $, *where* $P=D_{z}G(Q^{0},0)$ *is an M*-*matrix*, *and* Ω *is the biological feasible region*. *Hence* $E^{0}$ *is* (*GAS*) *if* $\mathcal{R}_{0}<1$.

#### Theorem 3

*The syphilis*-*free equilibrium of system* (2) *is* (*GAS*) *if* $\mathcal{R}_{0}<1$ *and unstable otherwise*.

#### Proof

We have to establish that conditions $(H_{1})$ and $(H_{2})$ hold when $\mathcal{R}_{0}<1$. For the uninfected population, we have

15 $$ F(Q,0)= \begin{pmatrix} \pi _{m}-\mu S_{m} \\ 0 \\ \pi _{f}-\mu S_{f} \\ 0 \end{pmatrix} . $$

Denoting by $Q\in \mathbb{R}^{6}$ the infected compartments in model (2), we have $G(Q,Z)=PZ-\hat{G}(Q,Z)$, where

$$ P= \begin{pmatrix} -(\gamma _{m}+\mu +\sigma _{m_{1}}) & 0 & 0 & \frac{\alpha _{f}S_{m}}{N} & \frac{\alpha _{f}S_{m}}{N} & \frac{\alpha _{f}S_{m}}{N} \\ \gamma _{m}& -(\beta _{m} +\mu +\sigma _{m_{2}}) & 0 & 0 & 0 & 0 \\ 0 & \beta _{1} & -(\mu +\sigma _{m_{3}}) & 0 & 0 & 0 \\ \frac{\alpha _{m}S_{f}}{N} & \frac{\alpha _{m}S_{f}}{N} & \frac{\alpha _{m}S_{f}}{N} & -(\gamma _{f}+\mu +\rho _{f_{1}}) & 0 & 0 \\ 0 & 0 & 0 & \gamma _{f} & -(\beta _{f}+\mu +\rho _{f_{2}}) & 0 \\ 0 & 0 & 0 & 0 & \beta _{f} & -(\mu +\rho _{f_{3}}) \end{pmatrix} . $$

Thus

16 $$ \hat{G}(Q, Z)= \begin{pmatrix} \hat{G}_{1}(Q, Z) \\ \hat{G}_{2}(Q, Z) \\ \hat{G}_{3}(Q, Z) \\ \hat{G}_{4}(Q, Z) \\ \hat{G}_{5}(Q, Z) \\ \hat{G}_{6}(Q, Z) \end{pmatrix} \begin{pmatrix} \alpha _{1}(I_{fp}+I_{fs}+L_{f})(1-\frac{S_{m}}{N}) \\ 0 \\ 0 \\ \alpha _{2}(I_{mp}+I_{ms}+L_{m})(1-\frac{S_{f}}{N}) \\ 0 \\ 0 \end{pmatrix} \begin{pmatrix} I_{mp} \\ I_{ms} \\ L_{m} \\ I_{fp} \\ I_{fs} \\ L_{f} \end{pmatrix}. $$

Since $S_{m} < N$ and $S_{f} < N$, we have $\hat{G}_{1}(Q,Z),\hat{G}_{2}(Q,Z),\hat{G}_{3}(Q,Z),\hat{G}_{4}(Q,Z), \hat{G}_{5}(Q,Z), \hat{G}_{6}(Q,Z)\geq 0$. The global stability of $Q^{0}= (\frac{\pi _{m}}{\mu },0,0,0,0,\frac{\pi _{f}}{\mu },0,0,0,0 )$ of the system $\frac{dQ}{dt}=F(Q^{0},0)$ is easy to verify. Therefore $Q^{0}$ is globally asymptotically stable if $\mathcal{R}_{0}<1$. This completes the proof. □

### Bifurcation analysis of the syphilis model

In this section, we adopt the techniques established in [7, 9, 16] to study the bifurcation analysis for the syphilis system (2). We apply center manifold theory [24] to the syphilis system (2) by taking $\mathcal{R}_{0}=1$ if and only if

$$ \alpha _{f}=\alpha _{f}^{*}= \frac{ (q_{1} q_{2} q_{3} q_{4} q_{5} q_{6} (\pi _{m}+\pi _{f}) )}{\psi ^{2}\alpha _{m}\pi _{m}\pi _{f}(\beta _{m}\gamma _{m}+\gamma _{m}q_{2}+q_{2}q_{3})(\beta _{f}\gamma _{2}+\gamma _{2}q_{6}+q_{5}q_{6})} . $$

We introduce a new set of variables for the syphilis model (2) for convenience sake by letting $x_{1} = S_{m}$, $x_{2} = I_{mp}$, $x_{3} = I_{ms}$, $x_{4} = L_{m}$, $x_{5} = R_{m}$, $x_{6} = S_{f}$, $x_{7} = I_{fp}$, $x_{8} = I_{fs}$, $x_{9} = L_{f}$, $x_{10} = R_{f}$, $x=(x_{1}, x_{2}, x_{3}, x_{4}, x_{5}, x_{6}, x_{7}, x_{8}, x_{9}, x_{10} )^{T}$, and $f=(f_{1}, f_{2}, f_{3}, f_{4}, f_{5}, f_{6}, f_{7}, f_{8}, f_{9}, f_{10})^{T}$. Thus we write model (2) in the form of the differential equation

$$ \frac{dx}{dt}=f(x,\alpha _{f}), $$

that is,

17 $$ \begin{aligned} \textstyle\begin{cases} f_{1}=\pi _{m}+\varphi _{m} x_{5}-\alpha _{f}\psi ( \frac{x_{7}+x_{8}+x_{9}}{x_{1}+x_{2}+x_{3}+x_{4}+x_{5}+x_{6}+x_{7}+x_{8}+x_{9}+x_{10}} )x_{1}-\mu x_{1}, \\ f_{2}=\alpha _{f}\psi ( \frac{x_{7}+x_{8}+x_{9}}{x_{1}+x_{2}+x_{3}+x_{4}+x_{5}+x_{6}+x_{7}+x_{8}+x_{9}+x_{10}} )x_{1}-\gamma _{m}{x_{2}}-\mu {x_{2}}-\sigma _{m_{1}}{x_{2}}, \\ f_{3}=\gamma _{m}{x_{2}}-\beta _{m}{x_{3}}-\sigma _{m_{2}}{x_{3}}-\mu {x_{3}}, \\ f_{4}=\beta _{m}{x_{3}}-\mu {x_{4}}-\sigma _{m_{3}}{x_{4}}, \\ f_{5}=\sigma _{m_{1}}x_{2}+\sigma _{m_{2}}x_{3}+\sigma _{m_{3}}x_{4}- \mu {x_{5}}-\varphi _{m}x_{5}, \\ f_{6}=\pi _{f}+\varphi _{f}{x_{6}}-\alpha _{m}\psi ( \frac{x_{2}+x_{3}+x_{4}}{x_{1}+x_{2}+x_{3}+x_{4}+x_{5}+x_{6}+x_{7}+x_{8}+x_{9}+x_{10}} )x_{6}-\mu {x_{6}}, \\ f_{7}=\alpha _{m}\psi ( \frac{x_{2}+x_{3}+x_{4}}{x_{1}+x_{2}+x_{3}+x_{4}+x_{5}+x_{6}+x_{7}+x_{8}+x_{9}+x_{10}} )x_{6}-\gamma _{f}{x_{7}}-\mu {x_{7}}-\rho _{f_{1}}x_{7}, \\ f_{8}=\gamma _{f}{x_{7}}-\beta _{f}{x_{8}}-\rho _{f_{2}}{x_{8}}-\mu {x_{8}}, \\ f_{9}=\beta _{f}{x_{8}}-\mu {x_{9}}-\rho _{m_{3}}{x_{9}}, \\ {} f_{10}=\rho _{f_{1}}x_{7}+\rho _{f_{2}}x_{8}+\rho _{f_{3}}x_{9}- \mu {x_{10}}-\varphi _{f}x_{10}. \end{cases}\displaystyle \end{aligned} $$

Computing the Jacobian matrix $J(E^{0}, \alpha _{f}) $ associated with (17) at syphilis-free equilibrium $E^{0} $ yields

$$ \begin{pmatrix} -r_{1} & 0 & 0 & 0 &\varphi _{1} & 0 & - \frac{\alpha _{f} \psi \pi _{m}}{\pi _{m}+\pi _{f}}& - \frac{\alpha _{f}\psi \pi _{m}}{\pi _{m}+\pi _{f}} & - \frac{\alpha _{f}\psi \pi _{m}}{\pi _{m}+\pi _{f}} & 0 \\ 0 & -r_{2} & 0 & 0 & 0 & 0 & \frac{\alpha _{f} \psi \pi _{m}}{\pi _{m}+\pi _{f}}& \frac{\alpha _{f}\psi \pi _{m}}{\pi _{m}+\pi _{f}}& \frac{\alpha _{f}\psi \pi _{m}}{\pi _{m}+\pi _{f}}& 0 \\ 0& \gamma _{m} & -r_{3} & 0 & 0 & 0 & 0 & 0 & 0 & 0 \\ 0& 0& \beta _{m} & -r_{4} & 0 & 0 & 0 & 0 & 0 & 0 \\ 0& \sigma _{m_{1}} & \sigma _{m_{2}} & \sigma _{m_{3}} & -r_{5} & 0 & 0 & 0 & 0 & 0 \\ 0& -\frac{\alpha _{f}\psi \pi _{f}}{\pi _{m}+\pi _{f}} & - \frac{\alpha _{f}\psi \pi _{f}}{\pi _{m}+\pi _{f}} & - \frac{\alpha _{f}\psi \pi _{f}}{\pi _{m}+\pi _{f}} & 0 & -r_{6} & 0 & 0 & 0 & \varphi _{f} \\ 0&\frac{\alpha _{m}\psi \pi _{f}}{\pi _{m}+\pi _{f}} & \frac{\alpha _{m}\psi \pi _{f}}{\pi _{m}+\pi _{f}} & \frac{\alpha _{m}\psi \pi _{f}}{\pi _{m}+\pi _{f}} & 0 & 0 & -r_{7} & 0 & 0 & 0 \\ 0& 0 & 0 & 0 & 0 & 0 & \gamma _{f} & -r_{8} & 0 & 0 \\ 0& 0 & 0 & 0 & 0 & 0 & 0 & \beta _{f} & -r_{9} & 0 \\ 0& 0 & 0 & 0 & 0 & 0 & \rho _{f_{1}} & \rho _{f_{2}} & \rho _{f_{3}} & -r_{10} \end{pmatrix} . $$

Considering the matrix $J(E^{0}, \alpha _{f})$, there exists a simple eigenvalue, and the remaining eigenvalues have negative real parts. As a result, it is possible to apply center and manifold theory to the syphilis model (17). We further compute the right and left eigenvectors of the matrix to get $w_{1}=\frac{\psi _{m}w_{5}}{r_{1}}- \frac{\alpha _{f}\psi \pi _{m}}{(\pi _{m}+\pi _{f})r_{1}}w_{7}- \frac{\alpha _{f}\psi \pi _{m}}{(\pi _{m}+\pi _{f})r_{1}}w_{8}- \frac{\alpha _{f}\psi \pi _{m}}{(\pi _{m}+\pi _{f})r_{1}}w_{9}$, $w_{3}=\frac{r_{1}}{r_{3}}w_{2}$, $w_{4}=\frac{\beta _{m}\gamma _{m}^{2}}{r_{4}r_{3}^{2}}w_{2}$, $w_{5}= ( \frac{\sigma _{m_{1}}}{r_{5}}+ \frac{\sigma _{m_{2}} \gamma _{m}}{r_{3}r_{5}}+ \frac{\sigma _{m_{3}} \beta _{m}\gamma _{m}}{r_{3}r_{4}r_{5}} )w_{2}$, $w_{6}=- ( \frac{\alpha _{m}\psi \pi _{m}}{(\pi _{m}+\pi _{f})r_{6}}+ \frac{\alpha _{m}\psi \pi _{m}\gamma _{m}}{(\pi _{m}+\pi _{f})r_{3}r_{6}}+ \frac{\alpha _{m}\psi \pi _{m} \beta _{m} \gamma _{m}}{(\pi _{m}+\pi _{f})r_{3}r_{4}r_{6}} )w_{2}+\frac{\varphi _{m}}{r_{6}}w_{10}$, $w_{7}= ( \frac{\alpha _{m}\psi \pi _{m}}{(\pi _{m}+\pi _{f})r_{7}}+ \frac{\alpha _{m}\psi \pi _{m}\gamma _{m}}{(\pi _{m}+\pi _{f})r_{3}r_{7}}+ \frac{\alpha _{m}\psi \pi _{m} \beta _{m} \gamma _{m}}{(\pi _{m}+\pi _{f})r_{3}r_{4}r_{7}} )w_{2}$, $w_{8}= ( \frac{\alpha _{m}\psi \pi _{m}}{(\pi _{m}+\pi _{f})r_{7}}+ \frac{\alpha _{m}\psi \pi _{m}\gamma _{m}}{(\pi _{m}+\pi _{f})r_{3}r_{7}}+ \frac{\alpha _{m}\psi \pi _{m} \beta _{m} \gamma _{m}}{(\pi _{m}+\pi _{f})r_{3}r_{4}r_{7}} )\frac{\gamma _{2}}{r_{8}}w_{2}$, $w_{9}= ( \frac{\alpha _{m}\psi \pi _{m}}{(\pi _{m}+\pi _{f})r_{7}}+ \frac{\alpha _{m}\psi \pi _{m}\gamma _{m}}{(\pi _{m}+\pi _{f})r_{3}r_{7}}+ \frac{\alpha _{m}\psi \pi _{m} \beta _{m} \gamma _{m}}{(\pi _{m}+\pi _{f})r_{3}r_{4}r_{7}} )\frac{\gamma _{2}\beta _{2}}{r_{8}r_{9}}w_{2}$, $w_{10}=\frac{\rho _{f_{1}}}{r_{10}}w_{7}+ \frac{\rho _{f_{2}}}{r_{10}}w_{8}+\frac{\rho _{f_{3}}}{r_{10}}w_{7}$, $w_{2}=w_{2} >0$, and $v_{1}=0$, $v_{3}= ( \frac{\beta _{m}\alpha _{m}\alpha _{f}\psi ^{2} \pi _{m}\pi _{f}}{(\pi _{m}+\pi _{f})^{2}r_{2}r_{4}r_{7}}+ \frac{\beta _{m}\beta _{f}\alpha _{m}\alpha _{f}\psi ^{2}\gamma _{f} \pi _{m}\pi _{f}}{(\pi _{m}+\pi _{f})^{2}r_{3}r_{4}r_{7}r_{8}r_{9}}+ \frac{\beta _{m}\alpha _{m}\alpha _{f}\psi ^{2}\gamma _{f} \pi _{m}\pi _{f}}{(\pi _{m}+\pi _{f})^{2}r_{3}r_{4}r_{7}r_{8}}+ \frac{\alpha _{m}\alpha _{f}\psi ^{2} \pi _{m}\pi _{f}}{(\pi _{m}+\pi _{f})^{2}r_{3}r_{7}}+ \frac{\alpha _{m}\alpha _{f}\psi ^{2} \pi _{m}\pi _{f}}{(\pi _{m}+\pi _{f})^{2}r_{3}r_{7}r_{8}}+ \frac{\beta _{f}\alpha _{m}\alpha _{f}\psi ^{2}\gamma _{f} \pi _{m}\pi _{f}}{(\pi _{m}+\pi _{f})^{2}r_{3}r_{7}r_{8}r_{9}} )v_{2}$, $v_{4}= ( \frac{\alpha _{m}\alpha _{f}\psi ^{2} \pi _{m}\pi _{f}}{(\pi _{m}+\pi _{f})^{2}r_{4}r_{7}}+ \frac{\beta _{f}\alpha _{m}\alpha _{f}\psi ^{2}\gamma _{f} \pi _{m}\pi _{f}}{(\pi _{m}+\pi _{f})^{2}r_{4}r_{7}r_{8}r_{9}}+ \frac{\alpha _{m}\alpha _{f}\psi ^{2}\gamma _{f} \pi _{m}\pi _{f}}{(\pi _{m}+\pi _{f})^{2}r_{4}r_{7}r_{8}} )v_{2}$, $v_{5}=0$, $v_{6}=0$, $v_{7}= ( \frac{\alpha _{f}\psi \pi _{m}}{(\pi _{m}+\pi _{f})r_{7}}+ \frac{\beta _{f}\alpha _{f}\psi \gamma _{f} \pi _{m}}{(\pi _{m}+\pi _{f})r_{7}r_{8}r_{9}}+ \frac{\alpha _{f}\psi \gamma _{f} \pi _{m}}{(\pi _{m}+\pi _{f})r_{7}r_{8}} )v_{2}$, $v_{8}= ( \frac{\alpha _{f}\psi \beta _{f} \pi _{m}}{(\pi _{m}+\pi _{f})r_{8}r_{9}}+ \frac{\alpha _{f}\psi \pi _{m}}{(\pi _{m}+\pi _{f})r_{8}} )v_{2}$, $v_{9}=\frac{\alpha _{f}\psi \pi _{m}}{(\pi _{m}+\pi _{f})r_{9}}v_{2}$, $v_{10}=0$, $v_{2}=v_{2} >0$, where $r_{1}=\mu $, $r_{2}=(\gamma _{m}+\mu +\sigma _{m_{1}})$, $r_{3}=(\beta _{m}+\mu +\sigma _{m_{2}})$, $r_{4}=(\mu +\sigma _{m_{3}})$, $r_{5}=(\mu +\varphi _{m})$, $r_{6}=\mu $, $r_{7}=(\gamma _{f}+\mu +\rho _{f_{1}})$, $r_{8}=(\gamma _{f}+\mu +\rho _{f_{2}})$, $r_{9}=(\mu +\rho _{f_{3}})$, and $r_{10}=(\mu +\varphi _{f})$.

Computing the bifurcation coefficients *a* and *b* after rigorous simplification yields $a= \frac{v_{2}w_{2}\alpha _{f} \mu }{\pi _{m}+\pi _{f}}+ \frac{v_{7}w_{2}\mu \alpha _{m}w_{7}}{\pi _{m}+\pi _{f}}>0$, $b=\frac{v_{7}w_{2}}{\pi _{m}+\pi _{f}}>0$.

Thus system (2) exhibits backward bifurcation.

### Sensitivity analysis of the syphilis model

In this section, we test the effect of system (2) parameters on the basic reproduction number $\mathcal{R}_{0}$ to ascertain the impact of these parameters on syphilis transmission. To get the sensitivity index, we partially differentiated $\mathcal{R}_{0}$ with respect to model (2) parameters. The formula used for the sensitivity analysis in this work, for example, in the case of $\pi _{m}$ is $\frac{\partial \mathcal{R}_{0}}{\partial \pi _{m}}\times \frac{\partial \pi _{m}}{\partial \mathcal{R}_{0}}$, is the same as used for all parameters of model (2). The result is presented in the Table 3.

We observe that the parameters $\pi _{m}$, $\pi _{f}$, $\alpha _{m}$, $\alpha _{f}$, *ψ*, $\gamma _{m}$, and $\gamma _{f}$ have positive sensitivity indices, which means that $\mathcal{R}_{0}$ increases with the parameter. The remaining parameters, $\beta _{m}$, $\beta _{f}$, $\sigma _{m_{1}}$, $\sigma _{m_{2}}$, $\rho _{m_{1}}$, and $\rho _{m_{2}}$ have negative values, which implies that $\mathcal{R}_{0}$ decreases for higher values of the parameters. For instance, the implication of the sensitivity index implies an increase (or decrease) of $\pi _{m}$ by a certain percentage, say, *y*% will result in an increase (or decrease) effect on the reproduction number by *y*%.

## Optimal control of the syphilis model

In this section, we present optimal control interventions for effective management of the syphilis infection. In an attempt to arrest the transmission of syphilis, we incorporated control interventions into system (2) to get

18 $$ \begin{aligned} \textstyle\begin{cases} \frac{dS_{m}}{dt} =\pi _{m}+\varphi _{m}{R_{m}}-\alpha _{f}\psi (1-u_{1}) (\frac{I_{fp}+I_{fs}+L_{f}}{N} )S_{m}-\mu {S_{m}}, \\ {} \frac{dI_{mp}}{dt} =\alpha _{f}\psi (1-u_{1}) ( \frac{I_{fp}+I_{fs}+L_{f}}{N} )S_{m}-\gamma _{m}{I_{mp}}-\mu {I_{mp}}- \sigma _{m_{1}}u_{2}{I_{mp}}, \\ {} \frac{dI_{ms}}{dt} =\gamma _{m}{I_{mp}}-\beta _{m}{I_{ms}}-\sigma _{m_{2}}u_{2}{I_{ms}}- \mu {I_{ms}}, \\ \frac{dL_{m}}{dt} =\beta _{m}{I_{ms}}-\mu {L_{m}}-\sigma _{m_{3}}u_{2}{L_{m}}, \\ {} \frac{dR_{m}}{dt} =\sigma _{m_{1}}u_{2}I_{mp}+\sigma _{m_{2}}u_{2}I_{ms}+ \sigma _{m_{3}}u_{2}L_{m}-\mu {R_{m}}-\varphi _{m}R_{m}, \\ {} \frac{dS_{f}}{dt} =\pi _{f}+\varphi _{f}{R_{f}}-\alpha _{m}\psi (1-u_{1}) (\frac{I_{mp}+I_{ms}+L_{m}}{N} )S_{f}-\mu {S_{f}}, \\ {} \frac{dI_{fp}}{dt} =\alpha _{f}\psi (1-u_{1}) ( \frac{I_{mp}+I_{ms}+L_{m}}{N} )S_{f}-\gamma _{f}{I_{f}p}-\mu {I_{f}p}- \rho _{f_{1}}u_{3}I_{fp}, \\ {} \frac{dI_{fs}}{dt} =\gamma _{f}{I_{f}p}-\beta _{f}{I_{fs}}-\rho _{f_{2}}u_{3}{I_{fs}}- \mu {I_{fs}}{}, \\ \frac{dL_{f}}{dt} =\beta _{f}{I_{fs}}-\mu {L_{f}}-\rho _{f_{3}}u_{3}{L_{f}}, \\ {} \frac{dR_{f}}{dt} =\rho _{f_{1}}u_{3}I_{fp}+\rho _{f_{2}}u_{3}I_{fs}+ \rho _{f_{3}}u_{3}L_{f}-\mu {R_{f}}-\varphi _{f}R_{f}. \end{cases}\displaystyle \end{aligned} $$

Basically, we present the objective functional *J* to investigate the optimal level of effort required to control the syphilis infection. We follow the techniques of [22] to formulate the objective functional, which is given by

19 $$ J(u)= \int _{0}^{t_{f}} \biggl[(A_{1} (I_{p}+I_{s}+L)+ \frac{1}{2}\bigl(B_{1} u_{1}^{2}+B_{2} u_{2}^{2}+B_{3} u_{3}^{2}\bigr) \biggr]\,dt, $$

where $I_{p}=(I_{mp}+I_{fp})$, $I_{s}=(I_{ms}+I_{fs})$, and $L=(L_{m}+ L_{f})$.

The factor $u_{1}$ is a control function representing prevention from syphilis infection through the use of condom and safe sex activity in both the male and female populations, $u_{2}$ is a control function representing treatment using antibiotic in male population, and $u_{3}$ is a control function representing treatment using antibiotic in female population. The use of condom and safe sex is aimed at reducing the transmission of syphilis from infected to susceptible individuals. $t_{f}$ is the final time, and the coefficients $A_{1}$, $B_{1}$, $B_{2}$, $B_{3}$ are positive weights to balance the factors. The aim is minimizing the number of males and females with primary stage syphilis $I_{p}$, the number of males and females with secondary stage syphilis $I_{s}$, and the number of males and females with latent stage syphilis *L* while we keep the cost of controls $u_{1}(t)$, $u_{2}(t)$, $u_{3}(t)$ at minimal level. Thus we seek optimal controls $u_{1}^{\ast }$, $u_{2}^{\ast }$, $u_{3}^{\ast }$ such that $J(u_{1}^{\ast }, u_{2}^{\ast }, u_{3}^{\ast })=\min_{u_{1},u_{2},u_{3}} \{ J(u_{1},u_{2},u_{3})\ni u_{1},u_{2},u_{3}\in U \} $, where *U* is the set of measurable functions from $[0, t_{f}]$ onto $[0, 1]$. The necessary conditions that an optimal control must satisfy were derived from Pontryagin’s maximum principle [32], and the existence of optimal control was derived from the adjoint variable of the state variables satisfying the following set of differential equations. This principle converts system (18) into a problem of minimizing pointwise a Hamiltonian *H* with respect to $(u_{1}, u_{2}, u_{3})$. The Hamiltonian is

20 $$ \begin{aligned} H&=A_{1} (I_{p}+I_{s}+L)+ \frac{1}{2}\bigl(B_{1} u_{1}^{2}+B_{2} u_{2}^{2}+B_{3} u_{3}^{2} \bigr) \\ &\quad{}+\lambda _{S_{m}} \biggl[\pi _{m}+\varphi _{m} R_{m}-(1-u_{1})\alpha _{f} \psi \biggl(\frac{I_{fp}+I_{fs}+L_{f}}{N} \biggr)S_{m}-\mu S_{m} \biggr] \\ &\quad{}+\lambda _{I_{mp}} \biggl[(1-u_{1})\alpha _{f}\psi \biggl( \frac{I_{fp}+I_{fs}+L_{f}}{N} \biggr)S_{m}-(\gamma _{m}+\mu +\sigma _{m_{1}} u_{2}) I_{mp} \biggr] \\ &\quad{}+\lambda _{I_{ms}}\bigl[\gamma _{m} I_{mp}-( \beta _{m}+\sigma _{m_{2}} u_{2}+ \mu ) I_{ms}\bigr] \\ &\quad{}+\lambda _{L_{m}}\bigl[\beta _{m} I_{ms}-( \mu +\sigma _{m_{3}} u_{2}) L_{m}\bigr] \\ {} &\quad{}+\lambda _{R_{m}}\bigl[\sigma _{m_{1}} u_{2} I_{mp}+\sigma _{m_{2}} u_{2} I_{ms}+\sigma _{m_{3}} u_{2} L_{m}-(\mu +\varphi _{m})R_{m}\bigr] \\ &\quad{}+\lambda _{S_{f}} \biggl[\pi _{f}+\varphi _{f} R_{f}-(1-u_{1})\alpha _{m} \psi \biggl(\frac{I_{mp}+I_{ms}+L_{m}}{N} \biggr)S_{f}-\mu S_{f} \biggr] \\ &\quad{}+\lambda _{I_{fp}} \biggl[(1-u_{1})\alpha _{m}\psi \biggl( \frac{I_{mp}+I_{ms}+L_{m}}{N} \biggr)S_{f}-(\gamma _{f}+\mu +\rho _{f_{1}} u_{3}) I_{fp} \biggr] \\ {} &\quad{}+\lambda _{I_{fs}}\bigl[\gamma _{f} I_{fp}-(\beta _{f} +\rho _{f_{2}} u_{3}+ \mu ) I_{fs}\bigr] \\ &\quad{}+\lambda _{L_{f}}\bigl[\beta _{f} I_{fs}-( \mu +\rho _{f_{3}} u_{3}) L_{f}\bigr] \\ &\quad{}+\lambda _{R_{f}}\bigl[\rho _{f_{1}} u_{3} I_{fp}+\rho _{f_{2}} u_{3} I_{fs}+ \rho _{f_{3}} u_{3} L_{f}-(\mu +\varphi _{f}) R_{f}\bigr], \end{aligned} $$

where $\lambda _{S_{m}}$, $\lambda _{I_{mp}}$, $\lambda _{I_{ms}}$, $\lambda _{L_{m}}$, $\lambda _{R_{m}}$, $\lambda _{S_{f}}$, $\lambda _{I_{fp}}$, $\lambda _{I_{fs}}$, $\lambda _{L_{f}}$, and $\lambda _{R_{f}}$ are the adjoint variables.

### Theorem 4

*Let* $u_{1}^{*}$, $u_{2}^{*}$, $u_{3}^{*}$ *be optimal controls*, *and let* $S_{m}$, $I_{mp}$, $I_{ms}$, $L_{m}$, $R_{m}$, $S_{f}$, $I_{fp}$, $I_{fs}$, $L_{f}$, *and* $R_{f}$ *be the solutions of the optimal control problem* (18)*–*(19) *that minimize* $J(u_{1},u_{2},u_{3})$ *over* *U*. *Then there exist adjoint variables* $\lambda _{S_{m}}$, $\lambda _{I_{mp}}$, $\lambda _{I_{ms}}$, $\lambda _{L_{m}}$, $\lambda _{R_{m}}$, $\lambda _{S_{m}}$, $\lambda _{I_{mp}}$, $\lambda _{I_{ms}}$, $\lambda _{L_{m}}$, $\lambda _{R_{m}}$ *satisfying*

21 $$ -\frac{d\lambda _{i}}{dt}=\frac{\partial H}{\partial i}, $$

*where* $S_{m}$, $I_{mp}$, $I_{ms}$, $L_{m}$, $R_{m}$, $S_{f}$, $I_{fp}$, $I_{fs}$, $L_{f}$, *and* $R_{f}$ *are the adjoint variables*, *and the controls* $u_{1}^{*}$, $u_{2}^{*}$, $u_{3}^{*}$ *obey the optimality conditions*

22 $$ \begin{gathered} u_{1}^{*} =\max \biggl\lbrace 0,\min \biggl(1,\\ \hphantom{u_{1}^{*} =} \frac{\frac{\alpha _{f} \psi S_{m}^{*}}{N^{*}}(I_{fp}^{*}+I_{fs}^{*}+L_{f}^{*})(\lambda _{I_{mp}}-\lambda _{S_{m}})+\frac{\alpha _{f}\psi S_{f}^{*}}{N^{*}}(I_{mp}^{*}+I_{ms}^{*}+L_{m}^{*})(\lambda _{I_{fp}}-\lambda _{S_{f}})}{B_{1}} \biggr) \biggr\rbrace , \\ u_{2}^{*} =\max \biggl\lbrace 0,\min \biggl(1,\\ \hphantom{u_{2}^{*} =} \frac{\sigma _{m_{1}} I_{mp}^{*}(\lambda _{R_{m}}-\lambda _{I_{mp}})+\sigma _{m_{2}} I_{ms}^{*}(\lambda _{R_{m}}-\lambda _{I_{ms}})+\sigma _{m_{3}} L_{m}^{*}(\lambda _{R_{m}}-\lambda _{ L_{m}})}{B_{2}} \biggr) \biggr\rbrace , \\ u_{3}^{*} =\max \biggl\lbrace 0,\min \biggl(1, \frac{\rho _{f_{1}} I_{fp}^{*}(\lambda _{R_{f}}-\lambda _{I_{fp}})+\rho _{f_{2}} I_{fs}^{*}(\lambda _{R_{f}}-\lambda _{I_{fs}})+\rho _{f_{3}} L_{f}^{*}(\lambda _{R_{f}}-\lambda _{L_{f}})}{B_{3}} \biggr) \biggr\rbrace . \end{gathered} $$

### Proof

To prove the theorem, we assessed the differentiated Hamiltonian functional at the optimal control to get the differentiable equations governing the adjoint variables. Hence

23 $$ \begin{gathered} \frac{d \lambda _{S_{m}}}{dt}= (1-u_{1}) \frac{\alpha _{f}\psi (I_{fp}^{*}+I_{fs}^{*}+L_{f}^{*})}{N^{*}}( \lambda _{S_{m}}-\lambda _{I_{mp}})+\mu \lambda _{S_{m}}, \\ \frac{d \lambda _{I_{mp}}}{dt}= (\lambda _{I_{mp}}-\lambda _{I_{ms}}) \gamma _{m}+\mu \lambda _{I_{mp}}+\sigma _{m_{1}} u_{2}(\lambda _{I_{mp}}- \lambda _{R_{m}})\\ \hphantom{\frac{d \lambda _{I_{mp}}}{dt}=}{}+ \frac{\alpha _{m}\psi (1-u_{1})S_{f}^{*}}{N^{*}}( \lambda _{S_{f}}-\lambda _{I_{fp}})-A_{1}, \\ \frac{d \lambda _{I_{ms}}}{dt}= (\lambda _{I_{ms}}-\lambda _{L_{m}}) \beta _{m}+\sigma _{m_{2}} u_{2}(\lambda _{I_{ms}}- \lambda _{R_{m}})+ \mu \lambda _{I_{ms}}\\ \hphantom{\frac{d \lambda _{I_{ms}}}{dt}=}{}+ \frac{\alpha _{m}\psi (1-u_{1})S_{f}^{*}}{N^{*}}(\lambda _{S_{f}}- \lambda _{I_{fp}})-A_{1}, \\ \frac{d \lambda _{L_{m}}}{dt}= \sigma _{m_{3}} u_{2}(\lambda _{L_{m}}- \lambda _{R_{m}})+\mu \lambda _{L_{m}}+ \frac{\alpha _{m} \psi (1-u_{1})S_{f}^{*}}{N^{*}}(\lambda _{S_{f}}- \lambda _{I_{fp}})-A_{1}, \\ \frac{d \lambda _{R_{m}}}{dt}= (\lambda _{R_{m}}-\lambda _{S_{m}}) \varphi _{m}+\mu \lambda _{R_{m}}, \\ \frac{d \lambda _{S_{f}}}{dt}= (1-u_{1}) \frac{\alpha _{m}\psi (I_{mp}^{*}+I_{ms}^{*}+L_{m}^{*})}{N^{*}}( \lambda _{S_{f}}-\lambda _{I_{fp}})+\mu \lambda _{S_{f}}, \\ \frac{d \lambda _{I_{fp}}}{dt}= \frac{\alpha _{f}\psi (1-u_{1})S_{m}^{*}}{N^{*}}( \lambda _{S_{m}}-\lambda _{I_{mp}})+(\lambda _{I_{fp}}-\lambda _{I_{fs}}) \gamma _{f}+\rho _{f_{1}} u_{3}(\lambda _{I_{fp}}- \lambda _{R_{f}})+ \mu \lambda _{I_{fp}}-A_{1}, \\ \frac{d \lambda _{I_{fs}}}{dt}= \frac{\alpha _{f}\psi (1-u_{1})S_{m}^{*}}{N^{*}}( \lambda _{S_{m}}-\lambda _{I_{mp}})+(\lambda _{I_{fs}}-\lambda _{L_{f}}) \beta _{f}+\rho _{f_{2}} u_{3}(\lambda _{I_{fs}}- \lambda _{R_{f}})+ \mu \lambda _{I_{fs}}-A_{1}, \\ \frac{d \lambda _{L_{f}}}{dt}= \frac{\alpha _{f} \psi (1-u_{1})S_{m}^{*}}{N^{*}}( \lambda _{S_{m}}-\lambda _{I_{mp}})+\rho _{f_{3}} u_{3}(\lambda _{ L_{f}}- \lambda R_{f})+\mu \lambda _{L_{f}}-A_{1}, \\ {} \frac{d \lambda _{R_{f}}}{dt}= (\lambda _{R_{f}}-\lambda _{S_{f}}) \varphi _{f}+\mu \lambda _{R_{f}} \end{gathered} $$

with transversality conditions:

24 $$ \textstyle\begin{cases} \lambda _{S_{m}}(t_{f})=\lambda _{I_{mp}}(t_{f})=\lambda _{I_{ms}}(t_{f})= \lambda _{L_{m}}(t_{f})=\lambda _{R_{m}}(t_{f})=\lambda _{S_{f}}(t_{f}) \\ \hphantom{\lambda _{S_{m}}(t_{f})}=\lambda _{I_{fp}}(t_{f})= \lambda _{I_{fs}}(t_{f})=\lambda _{L_{f}}(t_{f})= \lambda _{R_{f}}=0. \end{cases} $$

Also, the optimal functions $u_{1}^{*}$, $u_{2}^{*}$, and $u_{3}^{*}$ satisfy

$$ \frac{\partial H}{\partial u_{i}^{*}}=0,\quad i=1, 2, 3, $$

Therefore

$$\begin{aligned}& u_{1}^{\ast }= \frac{\frac{\alpha _{f} \psi S_{m}^{*}}{N^{*}}(I_{fp}^{*}+I_{fs}^{*}+L_{f}^{*})(\lambda _{I_{mp}}-\lambda _{S_{m}})+\frac{\alpha _{m} \psi S_{f}}{N^{*}}(I_{mp}^{*}+I_{ms}^{*}+L_{m}^{*})(\lambda _{I_{fp}}-\lambda _{S_{f}})}{B_{1}}, \\& u_{2}^{\ast }= \frac{\sigma _{m_{1}} I_{mp}^{*}(\lambda _{R_{m}}-\lambda _{I_{mp}})+\sigma _{m_{2}} I_{ms}^{*}(\lambda _{R_{m}}-\lambda _{I_{ms}})+\sigma _{m_{3}} L_{m}^{*}(\lambda _{R_{m}}-\lambda _{L_{m}})}{B_{2}}, \\& u_{3}^{\ast }= \frac{\rho _{f_{1}} I_{fp}^{*}(\lambda _{R_{f}}-\lambda _{I_{fp}})+\rho _{f_{2}} I_{fs}^{*}(\lambda _{R_{f}}-\lambda _{I_{fs}})+\rho _{f_{3}} L_{f}^{*}(\lambda _{R_{f}}-\lambda _{L_{f}})}{B_{3}}. \end{aligned}$$

In accordance with [22], based on typical control arguments involving the bound on the controls, we conclude that as a result of a priori boundedness of the state system and the adjoint system, we obtained the uniqueness of the optimality system (23)–(24). There is a restriction on the length of time interval $[0,t_{f}]$ so that we can guarantee the uniqueness of the optimality system [27]. □

## Numerical simulations

**Figure 2:**
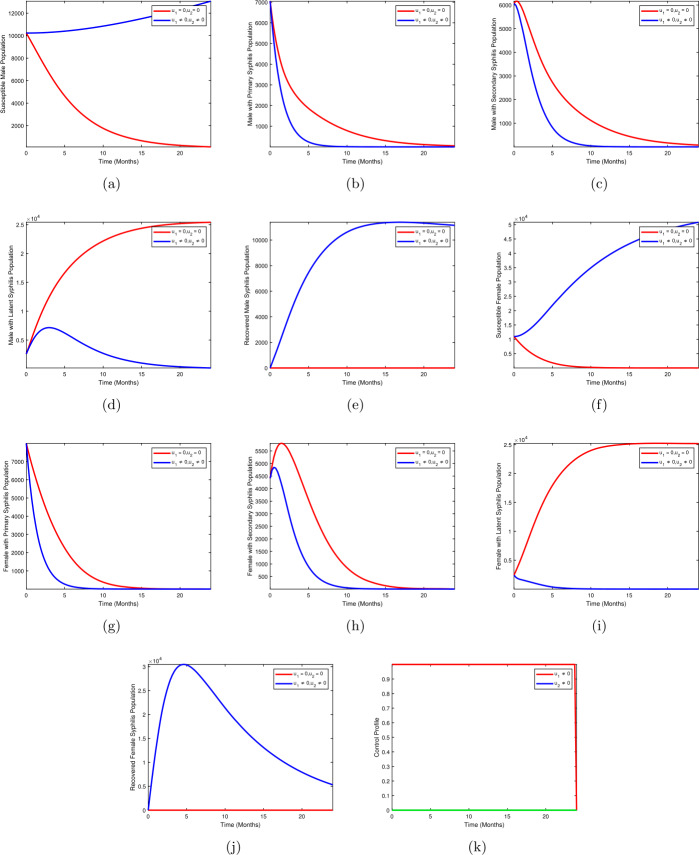
Use of condom + treatment of male with syphilis infection

**Figure 3:**
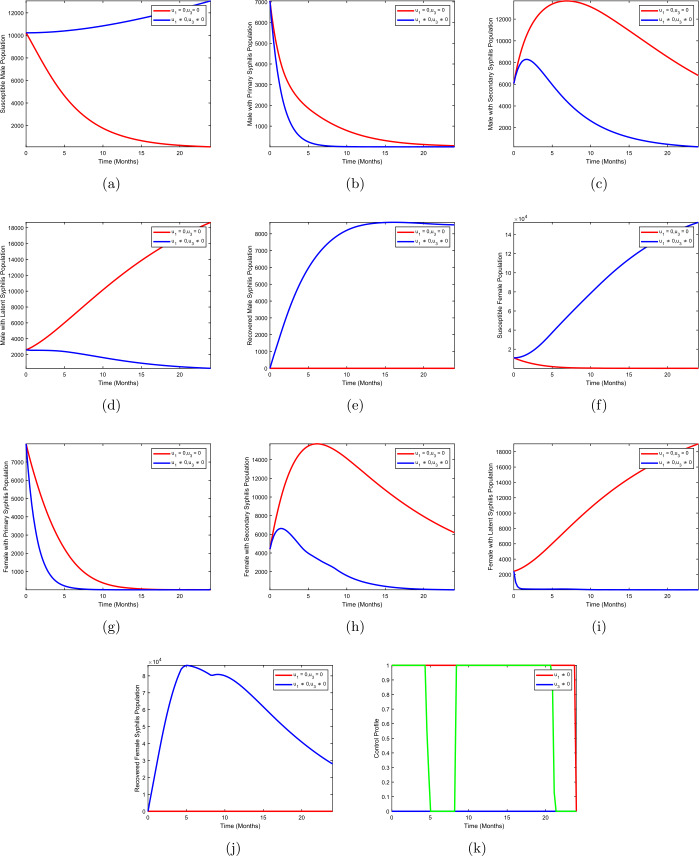
Use of condom + treatment of female with syphilis infection

**Figure 4:**
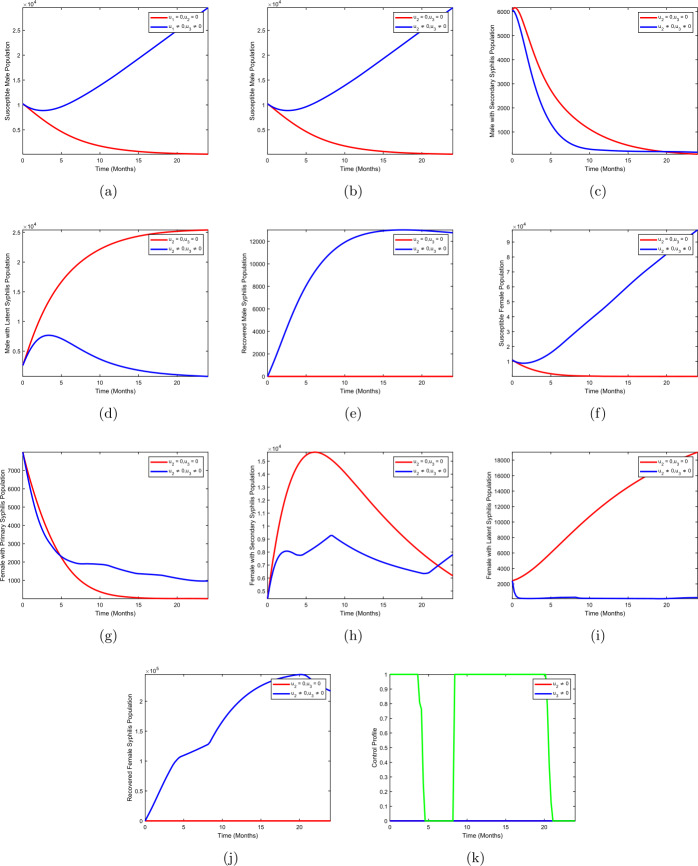
Treatment of male with syphilis infection + treatment of female with syphilis infection

**Figure 5:**
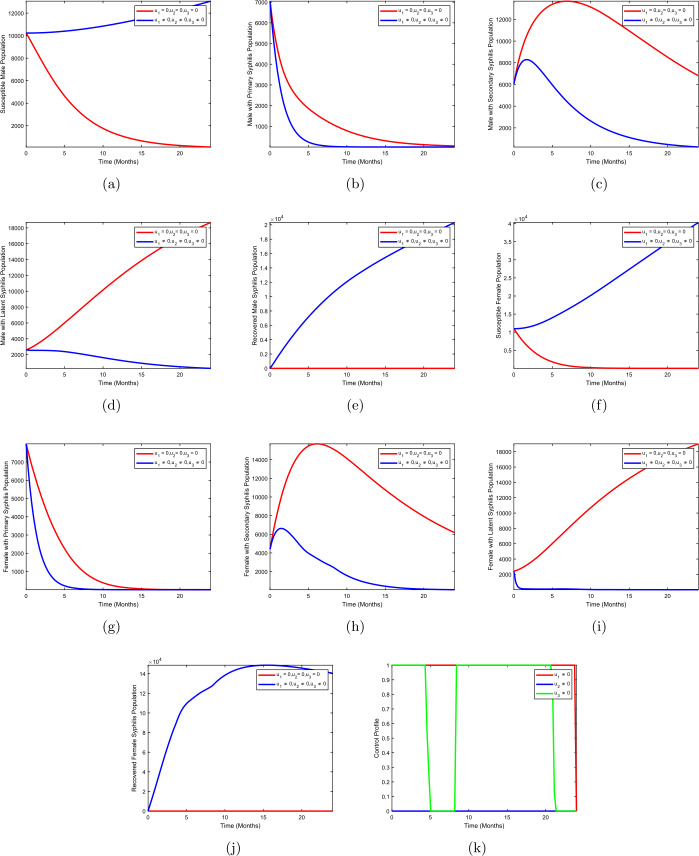
Use of condom + treatment of male with syphilis infection + treatment of female with syphilis infection

In this section, we investigate the impact of interventions on the transmission of syphilis in a population. The optimal control problem (18)–(19) is solved numerically following the techniques in [20], which uses the forward and backward Range–Kutta scheme. We use the variables and parameter values in Tables 2 and 4 to minimize the number of syphilis infections in both males and females. We implement the time level to be five years. The results are presented in Figs. 2–5 using the following strategies:

I. Strategy A: use of condom + treatment of male with syphilis infection.
II. Strategy B: use of condom + treatment of female with syphilis infection.
III. Strategy C: treatment of male with syphilis infection + treatment of female with syphilis infection.
IV. Strategy D: use of condom + treatment of male with syphilis infection + treatment of female with syphilis infection.

**Table 2:** Syphilis model parameters notation and values

Parameters	Description	Values	Source
$\pi _{m}$	Recruitment rate into susceptible male population	0.3	[29]
$\pi _{f}$	Recruitment rate into susceptible female population	0.45	[29]
$\alpha _{f}$	Transmission probability of female with syphilis infection	0.2	[15]
$\alpha _{m}$	Transmission probability of male with syphilis infection	0.5	[15]
$\gamma _{m}$	Progression rate from male with primary syphilis to male with secondary syphilis infection	0.01	[15]
$\gamma _{f}$	Progression rate from female with primary syphilis	0.627	[15]
*ψ*	Average number of sexual partner per unit time to female with secondary syphilis infection	2	[19]
$\beta _{m}$	Progression rate from male with secondary syphilis to male with latent syphilis infection	0.618	[15]
$\beta _{f}$	Progression rate from female with secondary syphilis to female individual with latent syphilis infection	0.618	[15]
$\varphi _{m}$	Rate of recovery from syphilis in infectious male	0.1	[29]
$\varphi _{f}$	Rate of recovery from syphilis in infectious female	0.1	[29]
$\sigma _{m_{1}}$	Treatment rate of male with primary stage syphilis	0.05	[29]
$\sigma _{m_{2}}$	Treatment rate of male with secondary stage syphilis	0.1	[29]
$\sigma _{m_{3}}$	Treatment rate of male with latent stage syphilis	0.2	[29]
$\rho _{f_{1}}$	Treatment rate of female with primary stage syphilis	0.05	[29]
$\rho _{f_{2}}$	Treatment rate of female with secondary stage syphilis	0.1	[29]
$\rho _{f_{3}}$	Treatment rate of female with latent stage syphilis	0.2	[29]
*μ*	Rate of natural death	5.48 × 10^−5^day^−1^	[42]
$A_{1}$	Weight coefficients for both infectious male and female syphilis	31	[38]
$B_{1}$	Relative cost for prevention and treatment in male and female individuals	0.5	[38]
$B_{2}$	Relative cost for prevention and treatment in male and female individuals	0.4	Assumed
$B_{3}$	Relative cost for prevention and treatment in male and female individuals	0.3	Assumed

**Table 3:** Sensitivity indices of syphilis model $\mathcal{R}_{0}$

Parameter	Description	Sensitivity Index
$\pi _{m}$	Recruitment rate into susceptible male population	1
$\pi _{f}$	Recruitment rate into susceptible female population	1
$\alpha _{f}$	Transmission probability of females with syphilis infection	0.5
$\alpha _{m}$	Transmission probability of males with syphilis infection	0.5
$\gamma _{m}$	Progression rate from male with primary syphilis to male with secondary syphilis infection	0.042548
$\gamma _{f}$	Progression rate from female with primary syphilis	0.37906
*ψ*	Average number of sexual partner per unit time to female with secondary syphilis infection	1
$\beta _{m}$	Progression rate from male with secondary syphilis to male with latent syphilis infection	−0.39089
$\beta _{f}$	Progression rate from female with secondary syphilis to female individual with latent syphilis infection	−0.0000013015
$\sigma _{m_{1}}$	Treatment rate of males with primary stage syphilis	−0.45322
$\sigma _{m_{2}}$	Treatment rate of males secondary stage syphilis	−0.0027407
$\rho _{f_{1}}$	Treatment rate females with primary stage syphilis	−0.25536
$\rho _{f_{2}}$	Treatment rate of females with secondary stage syphilis	−0.044735

**Table 4:** Initial values for the variables

Parameters	Value	Source
$S_{m}(0)$	10,230	Assumed
$I_{mp}(0)$	7048	[10]
$I_{ms}(0)$	6067	[10]
$L_{m}(0)$	2600	Assumed
$R_{m}(0)$	0	[28]
$S_{f}(0)$	10,960	Assumed
$I_{fp}(0)$	7998	[10]
$I_{fs}(0)$	4113	[10]
$L_{f}(0)$	2416	Assumed
$R_{f}(0)$	0	[28]

### Strategy A: use of condom + treatment of male with syphilis infection

In strategy A, we present the simulation of optimal control system (18) with condom use $(u_{1})$ as personal protection against infection and treatment of males with syphilis infection $(u_{2})$ are implemented, whereas treatment of female with syphilis infection $(u_{3})$ is set to zero. Figures 2(b–d) show the results of the implementation of strategy A. There is a significant difference in the population of males with primary syphilis infection $I_{mp}$, males with secondary syphilis infection $I_{ms}$, and males with latent syphilis infection $L_{m}$ when optimal use of condom $(u_{1}\neq 0)$ and treatment of males with syphilis infection $(u_{2}\neq 0)$ were compared to the population without optimal control strategy. Figures 2(g–i) show the results for females with primary syphilis infection $I_{mp}$, females with secondary syphilis infection $I_{ms}$, and females with latent syphilis infection $L_{m}$ when strategy A is applied. The population of females with primary syphilis infection $I_{fp}$, females with secondary syphilis infection $I_{fs}$, and females with latent syphilis infection $L_{f}$ drop when optimal use of condom $(u_{1}\neq 0)$ and treatment of males with syphilis infection $(u_{2}\neq 0)$ against the population without optimal control strategy. Figure 2(k) depicts the control profile for strategy A. We observed that the control curve for the use of condoms remains at the upper bound for almost the entire duration of the study period, that is, 24 months, whereas the curve for the treatment of males with syphilis infection remains at a lower bound for the duration of the study.

### Strategy B: use of condom + treatment of female with syphilis infection

This strategy shows the simulation of optimal control system (18) for condom use $(u_{1})$ as personal protection against infection and treatment of females with syphilis infection $(u_{3})$ while treatment of male with syphilis infection $(u_{2})$ is fixed at zero. Figures 3(b–d) show that there is a clear change in the population of males with primary syphilis infection $I_{mp}$, males with secondary syphilis infection $I_{ms}$, and males with latent syphilis infection $L_{m}$ when strategy B is effected compared to the population without optimal control strategy B. Figures 3(g–i) show the results for females with primary syphilis infection $I_{fp}$, females with secondary syphilis infection $I_{fs}$, and females with latent syphilis infection $L_{f}$ when strategy B is applied. We observed that the population of females with primary syphilis infection $I_{fp}$, females with secondary syphilis infection $I_{fs}$ and females with latent syphilis infection $L_{f}$ reduce when optimal use of condom $(u_{1}\neq 0)$ and treatment of males with syphilis infection $(u_{3}\neq 0)$ are implemented when compared to the case without control strategy. Figure 3(k) shows the control profile for strategy B. From the control curve we observed that the use of condoms remains at the upper bound for almost the entire duration of the study period, that is, 24 months, whereas the curve for the treatment of females with syphilis infection is unstable for the entire period of implementation of the strategy.

### Strategy C: treatment of male and female with syphilis infection

In strategy C, we use treatment of males with syphilis infection $(u_{2})$ and treatment of female with syphilis infection $(u_{3})$ to optimize the objective functional (19) while the use of condom $(u_{1})$ is set at zero. Figures 4(b–d) show that there is a drastic change in the population of males with primary syphilis infection $I_{mp}$, males with secondary syphilis infection $I_{ms}$, and males with latent syphilis infection $L_{m}$ when strategy C is effected compared to the population without optimal control strategy C. Figures 4(g–i) depict the results for females with primary syphilis infection $I_{fp}$, females with secondary syphilis infection $I_{fs}$, and females with latent syphilis infection $L_{f}$ when strategy B is applied. We observed that the population of females with primary syphilis infection $I_{fp}$, females with secondary syphilis infection $I_{fs}$, and females with latent syphilis infection $L_{f}$ diminishes when optimal treatment of males with syphilis infection $(u_{2}\neq 0)$ and treatment of females with syphilis infection $(u_{3}\neq 0)$ are implemented when compared to the case without control. Figure 4(k) shows the control profile for strategy C. We observed that the treatment of males with syphilis stays at a lower bound throughout the duration of the study period, whereas the curve for the treatment of females with syphilis infection oscillates throughout the period of implementation of the strategy.

### Strategy D: use of condom + treatment of male with syphilis infection + treatment of female with syphilis infection

Strategy D presents use of condom $(u_{1})$, treatment of males with syphilis infection $(u_{2})$, and treatment of female with syphilis infection $(u_{3})$ to optimize the objective functional (19). Figures 5(b–d) depict a more significant decrease in the population of males with primary syphilis infection $I_{mp}$, males with secondary syphilis infection $I_{ms}$, and males with latent syphilis infection $L_{m}$ when strategy C is effected compared to the population without optimal control strategy C. We also compared strategy D to other strategies and found out that the strategy shows a significant decline in the number of the infected population compared to other strategies. Figures 5(g–i) depict the results for females with primary syphilis infection $I_{fp}$, females with secondary syphilis infection $I_{fs}$, and females with latent syphilis infection $L_{f}$ when strategy C is implemented. We observed that the population of females with primary syphilis infection $I_{fp}$, females with secondary syphilis infection $I_{fs}$, and females with latent syphilis infection $L_{f}$ diminishes when optimal use of condom $(u_{1}\neq 0)$, treatment of males with syphilis infection $(u_{2}\neq 0)$, and treatment of females with syphilis infection $(u_{3}\neq 0)$ are implemented when compared to the case without control. Figure 5(k) depicts the control profile for strategy D. We observed that the curve for use of condom is at the upper bound for almost the entire period of the study (24 months), treatment of males with syphilis stays at lower bound throughout the duration of the study period, whereas the curve for the treatment of females with syphilis infection oscillates throughout the period of implementation of the strategy.

However, we realized that each of the four strategies explored in this study has positively demonstrated that the spread of syphilis can be halted once the desired group is targeted with given proper treatment. We equally noted that treating males can have an effect on females with syphilis infection and vice versa. Of the four strategies, strategy D, condom plus treatment in both the male and the female population, yielded the best result when compared to the use of treatment only strategy or condom plus treatment in male alone or condom plus treatment in female only strategies. This is supported by [21, 30, 37], respectively; treating the syphilis disease in its primary level and directing resources to the use of condoms plus treatment in a population would widely and immensely contribute in controlling the spread of syphilis.

## Conclusion

In this research, we proposed and studied a mathematical sex-structured syphilis model with three stages of infection and three control strategies, and loss of immunity. We assumed a constant control for the control parameters in the analytical solution. The positivity of the solution was proved, and the system of nonlinear differential equations is found to be biologically and mathematically well-posed. We obtained the basic reproduction number using the next-generation method. The syphilis-free and syphilis-present equilibria were established. The syphilis-free equilibrium is locally asymptotically stable when $\mathcal{R}_{0}<1$ and unstable when $\mathcal{R}_{0}>1$. The global stability of syphilis-free equilibrium is proved to be globally asymptotically stable when the associated reproduction number $\mathcal{R}_{0}<1$. This implies that the disease will completely die out in a stable equilibrium, whereas it will persist and become endemic in an unstable equilibrium. We used Pontryagin’s maximum principle to investigate the optimal level required to curtail the spread of syphilis in a population. Numerical results show that the best strategy for control of syphilis transmission is strategy D, the combination of condom usage for the prevention and treatment of infected male and female population.

## Data Availability

Not applicable.
